# Cytokine profile of anti-spike CD4^+^T cells predicts humoral and CD8^+^T cell responses after anti-SARS-CoV-2 mRNA vaccination

**DOI:** 10.1016/j.isci.2024.110441

**Published:** 2024-07-02

**Authors:** Nadine Benhamouda, Anissa Besbes, Rebecca Bauer, Nesrine Mabrouk, Gauthier Gadouas, Corinne Desaint, Lucie Chevrier, Maeva Lefebvre, Anne Radenne, Marie Roelens, Béatrice Parfait, Daniela Weiskopf, Alessandro Sette, Nadège Gruel, Marie Courbebaisse, Victor Appay, Stephane Paul, Guy Gorochov, Jacques Ropers, Said Lebbah, Jean-Daniel Lelievre, Ludger Johannes, Jonathan Ulmer, David Lebeaux, Gerard Friedlander, Xavier De Lamballerie, Patrice Ravel, Marie Paule Kieny, Fréderic Batteux, Christine Durier, Odile Launay, Eric Tartour

**Affiliations:** 1 Department of Immunology, Hôpital Européen Georges-Pompidou, Hôpital Necker Department of Immunology, Paris, France; 2 Université Paris Cité, INSERM U970, PARCC, Department of Immunology, Hôpital Européen Georges-Pompidou, Hôpital Necker Department of Immunology, Paris, France; 3 INSERM SC10-US019, Villejuif, France; 4 Bioinformatics and Cancer System Biology Team, IRCM-INSERM U1194, Institut de Recherche en Cancerologie de Montpellier, Montpellier, France; 5 Université Paris Cité, INSERM, CIC 1417, F-CRIN, Innovative Clinical Research Network in Vaccinology (I-REIVAC), APHP, CIC Cochin Pasteur, Hôpital Cochin, Paris, France; 6 Université Paris Cité, INSERM U1016 Insitut Cochin, Hôpital Cochin, APHP, Centre Service d’immunologie Biologique, Paris, France; 7 Service de maladies infectieuses et tropicales, Centre de prévention des maladies infectieuses et transmissibles CHU de Nantes, Nantes, France; 8 Unité de Recherche Clinique des Hôpitaux Universitaires Pitié Salpêtrière-Hôpitaux Universitaires Pitié Salpêtrière - Charles Foix, APHP, Paris, France; 9 Centre de ressources Biologiques, Hôpital Cochin, APHP, Paris, France; 10 Center for Infectious Disease and Vaccine Research, La Jolla Institute for Immunology (LJI), La Jolla, CA, USA; 11 Department of Medicine, School of Medicine in Health Sciences, University of California, San Diego (UCSD), La Jolla, CA, USA; 12 Department of Medicine, Division of Infectious Diseases and Global Public Health, University of California, San Diego (UCSD), La Jolla, CA, USA; 13 INSERM U830, Équipe Labellisée Ligue Nationale Contre le Cancer, Diversity and Plasticity of Childhood Tumors Lab, Centre de Recherche, Institut Curie, Université PSL, Paris, France; 14 Department of Translational Research, Centre de Recherche, Institut Curie, Université PSL, Paris, France; 15 Faculté de Médecine, Université Paris Cité, Paris, France; 16 Explorations fonctionnelles rénales, Physiologie, Hôpital Européen Georges-Pompidou, APHP, Paris, France; 17 Université de Bordeaux, CNRS UMR 5164, INSERM ERL 1303, ImmunoConcEpT, 33000 Bordeaux, France; 18 International Research Center of Medical Sciences, Kumamoto University, Kumamoto, Japan; 19 Centre International de Recherche en Infectiologie, Team GIMAP, Université Jean Monnet, Université Claude Bernard Lyon, INSERM, CIC 1408 INSERM Vaccinology, Immunology Department, iBiothera Reference Center, University Hospital of Saint-Etienne, Saint-Etienne, France; 20 Sorbonne Université, INSERM, Centre d'Immunologie et des Maladies Infectieuses, APHP, Hôpital Pitié-Salpêtrière, Paris, France; 21 Unité de Recherche Clinique des Hôpitaux Universitaires Pitié Salpêtrière –Hôpitaux Universitaires Pitié Salpêtrière- Charles Foix, APHP, Paris, France; 22 Vaccine Research Institute, Créteil, France; 23 INSERM U955, Université Paris-Est Créteil, Créteil, France; 24 Groupe Henri-Mondor Albert-Chenevier, APHP, Créteil, France; 25 Cellular and Chemical Biology Unit, U1143 INSERM, UMR3666 CNRS, Institut Curie, Centre de Recherche, Université PSL, Paris, France; 26 Université Paris Cité, Service de maladies infectieuses Hôpital Saint Louis/Lariboisère APHP, INSERM, Paris, France; 27 Department of « Croissance et Signalisation », Institut Necker Enfants Malades, INSERM U1151, CNRS UMR 8253, Université de Paris Cité, Paris, France; 28 Unité des Virus Émergents, UVE: Aix-Marseille Université, IRD 190, INSERM 1207 Marseille, France; 29 Institut National de la Santé et de la Recherche Médicale, INSERM, Paris, France

**Keywords:** Health sciences, Immunity, Virology, Mathematical biosciences, Machine learning

## Abstract

Coordinating immune responses – humoral and cellular – is vital for protection against severe Covid-19. Our study evaluates a multicytokine CD4^+^T cell signature’s predictive for post-vaccinal serological and CD8^+^T cell responses. A cytokine signature composed of four cytokines (IL-2, TNF-α, IP10, IL-9) excluding IFN-γ, and generated through machine learning, effectively predicted the CD8^+^T cell response following mRNA-1273 or BNT162b2 vaccine administration. Its applicability extends to murine vaccination models, encompassing diverse immunization routes (such as intranasal) and vaccine platforms (including adjuvanted proteins). Notably, we found correlation between CD4^+^T lymphocyte-produced IL-21 and the humoral response. Consequently, we propose a test that offers a rapid overview of integrated immune responses. This approach holds particular relevance for scenarios involving immunocompromised patients because they often have low cell counts (lymphopenia) or pandemics. This study also underscores the pivotal role of CD4^+^T cells during a vaccine response and highlights their value in vaccine immunomonitoring.

## Highlights

• After vaccination, cytokine profile of CD4^+^T cells predicts CD8^+^T cell response
• IP-10, IL-2, IL-9 and TNFα producing CD4^+^T cells correlated with CD8^+^T cell response
• IL-21 producing CD4^+^T cells correlated with humoral response
• CD4^+^T cells orchestrate the CD8^+^T cell and humoral responses after vaccination

## Graphical abstract

**Figure:**
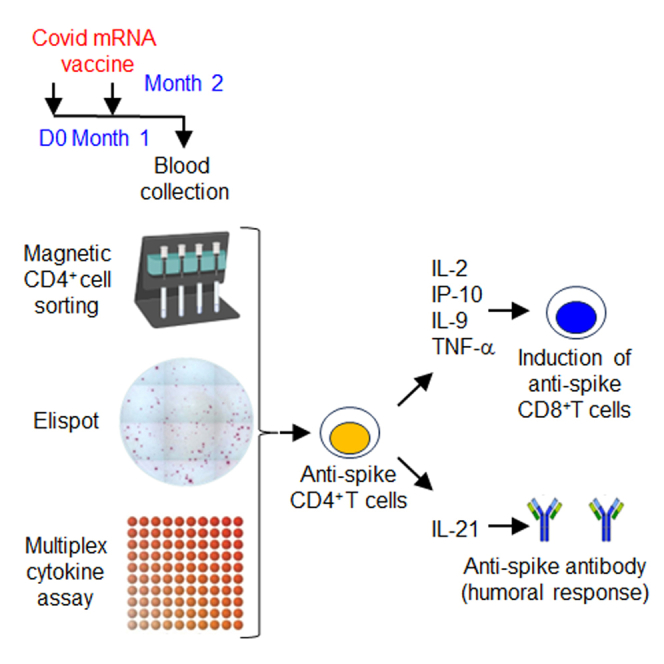

## Introduction

During COVID-19, neutralizing antibodies are mainly responsible for the prevention of infection, while T cells can reduce severe disease^1^ and partially control infection. More precisely, an effective immune response against SARS-CoV-2 appears to require early immune reactions, especially CD4^+^T cells,^2^^,^^3^^,^^4^^,^^5^ CD8^+^T cells,^6^ or both,^7^ along with Type I IFN production.^8^^,^^9^^,^^10^^,^^11^ While the role of neutralizing antibodies as a surrogate marker of protection is well accepted, the involvement of T cells in disease control has only recently been explored. Preclinical results demonstrated a substantial reduction in the ability of antibodies to control the viral infection in CD8^+^T cell-depleted vaccinated macaques.^12^ Moreover, several patients with inherited or treatment-induced B cell deficiencies have been reported to recover from COVID-19 in the absence of neutralizing antibodies.^13^^,^^14^^,^^15^ A recent study in patients with various immunosuppressive diseases vaccinated against the Sars-CoV-2 reported that lower serological or T cell responses were associated with hospitalization or death.^16^ Multiple studies suggest that coordinated early action by these immune components, often impaired in the elderly,^2^ is essential for infection control.

Another significant finding concerns immune response variability among patients after SARS-CoV-2 infection or vaccination. After infection, antibody responses to SARS-CoV-2 exhibited a 1,000-fold range in levels,^14^ alongside the spectrum of SARS-CoV-2-specific CD4^+^T cell and CD8^+^T cell responses.^2^^,^^17^ Around 30% of patients have undetectable anti-SARS-CoV-2 CD8^+^T cells,^18^ while circulating SARS-CoV-2 neutralizing antibody titers were low in a substantial fraction of recovered COVID-19 cases.^19^^,^^20^^,^^21^ This variability in response is similarly observed after vaccination, where the strength of CD8^+^T cell responses varies among individuals and studies.^22^^,^^23^^,^^24^ While most of the patients experienced antibody induction, their titers decreased over time. Various factors can explain this immune heterogeneity such as age, comorbidities (diabetes, cancer …), immunosuppressive medication, alcohol consumption, or preexisting antiviral immunity.^25^^,^^26^^,^^27^

To gain a better understanding of vaccine response and its correlation with immune response and disease control at the individual level, accurate assessment of these different immune elements becomes crucial. Depending on the study, antibody titers may correlate^2^^,^^22^ or may not correlate with the magnitude of the memory T cell response.^19^^,^^28^ This variability may be influenced by factors such as the timing of the analysis and the qualitative aspects of the antibodies and TH cells selected for this correlation. It suggests that simple serodiagnostic tests might not be reliably indicative of the cellular immune compartment. Therefore, such tests offer only a limited picture of the overall immune response.

Unfortunately, monitoring specific CD8^+^T cells proves challenging. They recognize a complex of an HLA class I molecule binding an 8-10-mer peptide, resulting in an extended repertoire for each viral variant. Detecting CD8^+^T cells can be detected by the conventional techniques using 15-mer peptide pools, spanning the entire viral protein sequence. However, sensitivity significantly improves with optimal 8-10-mer peptides, which are far more complex to identify. In contrast, CD4^+^T cells are much more straightforward to evaluate, as they recognize a complex of HLA class II molecules associated with a peptide ranging from 13 to 25-mers that is less constrained than the optimal peptide size for CD8^+^T cells. Therefore, a 15-mer peptide pool covering the complete sequence of the targeted protein is sufficient for accurately identifying specific CD4^+^ T cells.

Moreover, several functional subpopulations of CD4^+^T cells have been described according to their cytokine polarization. For example, Th1 CD4^+^T cells are characterized by the production of certain cytokines (IL-2, IFN-γ, IP10, TNF-α), primary promoting CD8^+^ T cell responses. In contrast, follicular helper (Tfh) CD4^+^T cells, defined by their production of IL-21, facilitate optimal B cell responses, germinal center activity, and antibody responses.^29^^,^^30^^,^^31^^,^^32^ In some viral infections, the quality of antigen-specific CD4^+^T cell responses, influenced by particular or several cytokine(s) or phenotypic profile(s), can outweigh their quantity, in conferring protection against reinfection or pathogen reactivation.^33^^,^^34^ Considering CD4^+^T cells as orchestrators of humoral and cellular responses, we explored here the hypothesis that multifunctional SARS-CoV-2-specific CD4^+^T cells could serve as surrogate predictive markers for inducting humoral response and specific CD8^+^T cells post-vaccination. This study aims to measure the multicytokine CD4^+^T cell response using the EliSpot and Luminex assays, covering distinct CD4^+^T cell subpopulations, in order to evaluate the test’s ability to predict both serological and CD8^+^T cell responses in patients immunized with the BNT162b2 mRNA vaccine.

## Results

### Characterization of the CD4^+^T cell assay to detect the vaccine induced response against the spike from Sars-CoV-2

After the enrichment step with CD4 magnetic beads, we observed a significant increase in the percentage of CD4^+^T cells before sorting, rising from 75-78% to 98–99%, after gating on live CD3^+^ T lymphocytes (Figures S1A and S1B). Simultaneously, the percentage of CD8^+^T lymphocytes increased from 18-20% to 89–92% after the elution of cells not retained on the beads (Figures S1A and S1C).

We then investigated the presence of cells other than T lymphocytes after enrichment on CD4 beads, which could potentially serve as antigen-presenting cells in addition to T cells. While T lymphocytes can contribute to presenting antigens to other T lymphocytes,^35^ the presence of antigen-presenting cells such as monocytes or B lymphocytes capable of reactivating a memory T lymphocyte response would be advantageous for this test.^36^^,^^37^^,^^38^

As expected, given that monocytes can express low levels of the CD4 molecule, we found that after purification, CD4 monocytes expressing the HLA-DR molecule and CD86 (the ligand for the CD28 costimulatory molecule) persist in our sample (Figures S2A–S2D). In our test, antigenic presentation of 15-mer peptides can, therefore, be performed by both monocytes and CD4^+^T cells, which also express HLA-DR (Figure S2D). Additionally, our analysis showed that in addition to the non-retained CD8^+^T cells eluted from the beads, this eluate also contains monocytes and B lymphocytes (Figures S2E and S2F), capable of presenting the optimal 8-9mer peptides selected for detecting of spike from Sars-CoV-2 by CD8^+^T cells.

In a final experiment, we compared the sensitivity of our assay to the use of PBMC from four patients vaccinated with BNT 162b2 mRNA. Our results demonstrated that the enrichment of CD4^+^T cells by magnetic beads increases the assay’s sensitivity for detecting CD4^+^T cells producing IFNγ against the pool of spike-derived peptides compared with PBMC in the four patients tested (Figures S3A and S3B). It’s noteworthy that when using PBMC, we cannot distinguish the cellular origin of IFNγ production (CD4 or CD8^+^T cells), which reinforces the increased sensitivity of CD4^+^T cell detection after sorting, where only CD4^+^T cells, and not CD8^+^T cells, produce IFNγ.

### Profile of cytokine response produced by CD4^+^T cell after vaccination

**Figure 1:**
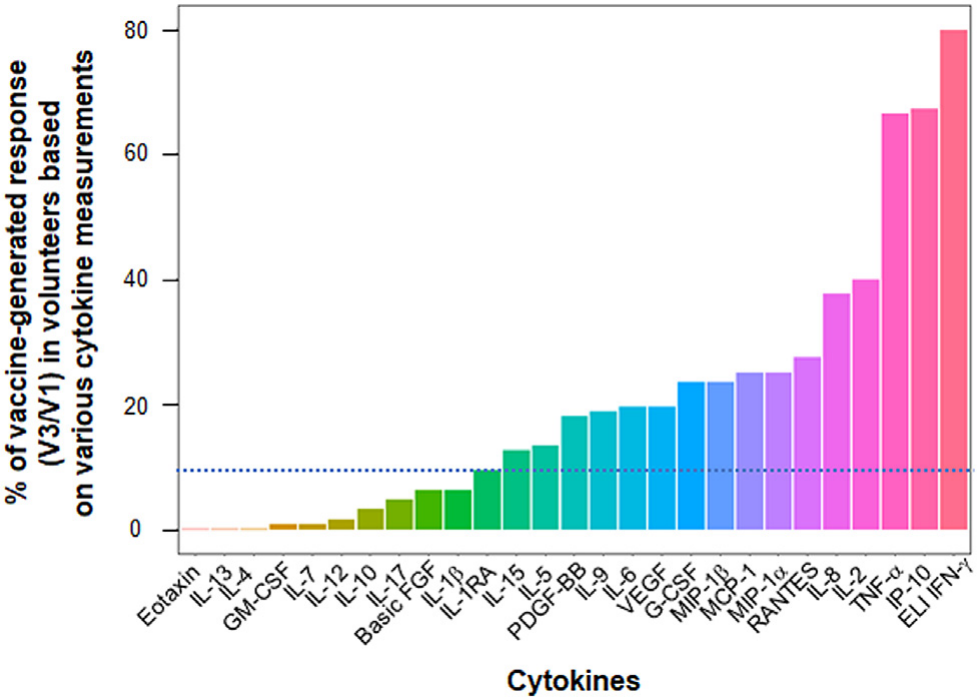
Percentage of vaccine-generated anti-spike CD4^+^T cell response depending on the cytokine measured A total of 128 patients, including 76 who were pre-infected and 52 who were uninfected, received vaccination. Non-pre-infected volunteers received BNT162b2 vaccine (30 μg) at V1 (D0) and V2 (D29), while pre-infected volunteers received only one dose of vaccine at V1. At V3, which is one month after the second vaccination or 2 months after the only 1^st^ vaccination depending on their infection status, the patients' CD4^+^T cells were sorted and sensitized *in vitro* with a megapool of overlapping peptides covering the S1 protein and another pool for the S2 protein. An ELIspot (ELI) IFNγ assay and a 27-cytokine Luminex assay, were then performed after 24 or 48 h of incubation, respectively. The Luminex assay was used with supernatants of ELIspot IFNγ not coated with anti-IFNγ antibodies. The frequency of vaccine response for each cytokine, as determined by the V3/V1 ratio ≥2, and a concentration of the cytokine ≥10 pg/mL (after background subtraction when cells were sensitized with medium) is shown. The threshold for vaccine response detection for a given cytokine is indicated by the dotted line at 10% frequency.

Out of 145 participants selected in CovicompareP, 128 were evaluable (distribution by groups in Table S1). A total of 26 different cytokines were measured via Luminex in the culture supernatant of sorted CD4^+^T cells, sensitized with two pools S1 and S2 of spike-derived peptides, in 128 patients who received the BTN162b2 mRNA vaccine at one month after the second dose (V3). Given a lower sensitivity of the Luminex for detection IFN-γ in this study, IFN-γ was measured by ELISpot, which served as our reference method. Sixteen cytokines produced by CD4^+^T cells facilitated the detection of a vaccine response against spike-derived peptides after two immunizations with the BTN162b2 vaccine in at least 10% of cases (Figure 1). In terms of the frequency positivity, these cytokines ranked as follows: IFN-γ, IP10, TNF-α (all positive in >65% of patients), followed by IL-2 (40%), IL-8, Rantes, MIP1-α, MCP1, MIP-1b G-CSF, VEGF, IL-6, IL-9, PDGF-β, IL-5, and IL-15. When comparing cytokines levels before (V1) and after (V3) vaccination, IFN-γ, IP-10, TNF-α, and IL-2 were the four predominant cytokines in terms of response intensity, with a median response induction factor (V3/V1 ratio) greater than 2 (Table S2). Other cytokines exhibited a vaccine response frequency below 10% (eotaxin, IL-13, IL-4, GM-CSF, IL-7, IL-12, IL-10, IL-17, basic FGF, IL-1-β, IL-1RA; Figure 1; Table S2). We did not pursue the analysis of these cytokines in the remainder of the work. Therefore, the vaccine response polarization appeared to be primarily Th1 and Th9, without the involvement of Th2 or Th17 responses. We also showed that there is little difference in the % of positive vaccine responses for a given cytokine when selecting the total population (Figure 1) or the pre-infected population (Figure S4 Right) or the non-pre-infected population (Figure S4 Left). Thus 14 cytokines are identical to whatever the population selected (Figure 1; Figure S4). Two cytokines, IL-5 and IL-15 are detectable in the total and pre-infected population but not in the non-pre-infected population with this 10% positive vaccine response threshold. One cytokine, IL-1ra is detected in the total and non-pre-infected population but not in the infected population.

We found no correlation between the cytokines V3/V1 ratios and the age of volunteers in the whole population, nor did we find any correlation with the infection severity in the pre-infected (PI) group (Figure S5A). Among individuals aged over 75 years, the production of no more than 10 cytokines was observed, regardless of whether they had been infected or not. In contrast, whereas 14% and 19% of subjects aged under 75 in the non-pre-infected (NPI) and PI groups, respectively, had the capability to produce 10 or more cytokines derived from anti-SARS-CoV-2 CD4^+^T cells (Figure S5B).

### Difference in cytokine profile from CD4^+^T cells between pre-infected and non-pre-infected individuals

**Figure 2:**
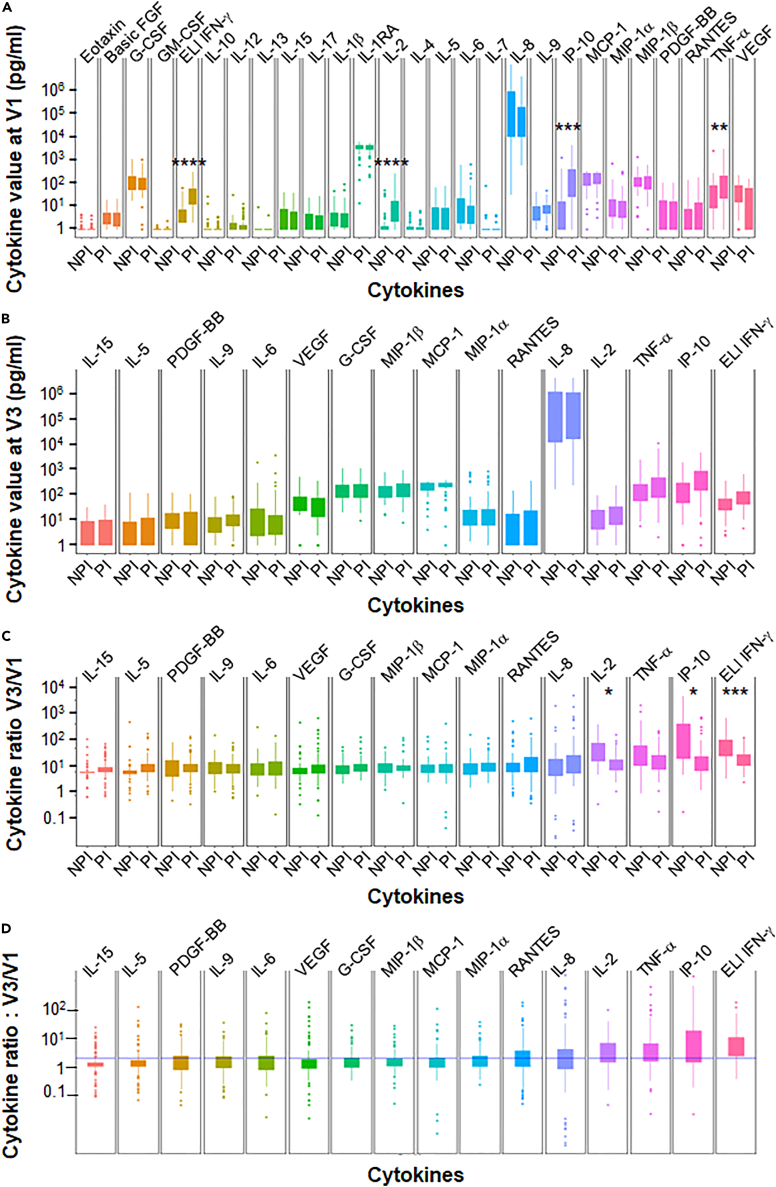
Difference in the induction of CD4^+^T cell vaccine response and the detection of T cell responses based on cytokine assays (A) Anti-spike multicytokine CD4^+^T cell responses were measured prior to vaccination (V1) in pre-infected (PI) (*n* = 76) and non-pre-infected (NPI) (*n* = 52) volunteers. (B) The absolute value of ELISpot IFNγ and cytokines assay were measured one month after the second BNT162b2 vaccination (V3) for non-pre-infected volunteers and after only one dose of vaccination for pre-infected participants at the same time. (C) The vaccine response based on CD4^+^T cell cytokines profile and the V3/V1 ratio was calculated for the same set of volunteers. (D) The ratio between V3 and V1 is shown regardless of infection status. Statistical differences, determined using the Wilcoxon test with FDR correction, are shown between the pre-infected and non-infected groups for each cytokine. Data are represented as mean ± SEM ∗: *p* ≤ 0.05; ∗∗: *p* ≤ 0.01; ∗∗∗: *p* ≤ 0.001.

When cytokine production in CD4^+^T cells sensitized with a specific pool of spike-derived peptides was analyzed prior to the first BTN162b2 vaccination, PI individuals (*n* = 76) showed a significantly higher concentration of IFN-γ, IL-2, IP10, and TNF-α compared to NPI subjects (Figure 2A). However, the other cytokines studied were not affected. The mean time between infection in PI subjects and the first vaccination was 9.3 months, indicating some persistence of the Th1 response after infection with SARS-CoV-2 (Wuhan index strain). However, at one month after the second vaccination for NPI (D57), PI individuals who received only one dose of vaccine did not show greater cytokine production than NPI individuals (Figure 2B).

The induction ratio of cytokines produced after vaccination was also not greater for PI individuals than for NPI individuals (Figure 2C). There was even a lower ratio in patients with PI for IFN-γ, IL-2, and IP10, and a trend for TNF-α, probably due to a higher basal level of these cytokines in those patients (Figure 2A).

### Value of this cytokine profile for increasing the sensitivity to detect antigen specific CD4^+^T lymphocyte response

**Figure 3:**
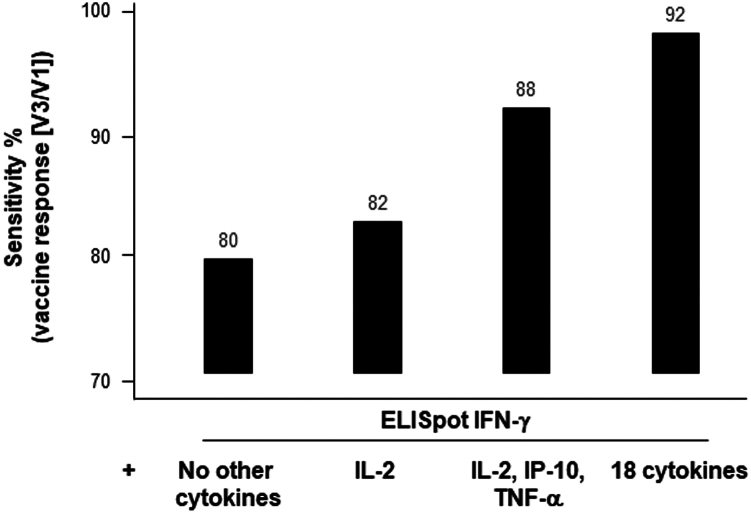
Enhancement in sensitivity of the anti-spike CD4^+^T cell assay through multiple cytokines detection A comparison of the CD4^+^T cell vaccine responses (V3/V1) was performed using either a positive ELISpot IFNγ assay alone (without cytokine) or in combination with the detection of IL-2 or 3 cytokines (IL-2, IP-10, TNFα), or 18 cytokines via Luminex (*n* = 128). The respective sensitivity of the different tests is shown on the histograms.

The ELISpot IFN-γ method demonstrated a sensitivity of approximately 80% in detecting a vaccine response (ratio V3/V1 ≥ 2) across the whole population (Figure 3). This sensitivity increased to 88% when focusing on the top four cytokines (IFN-γ, IL-2, TNF-α, IP-10) identified in the first part of our study. The inclusion of four additional cytokines (MCP-1, MIP1-α, MIP1-β, IL-15) further raised the sensitivity to 92%. Among the 19 other tested cytokines, none significantly contributed to the sensitivity of this model. Unfortunately, we were unable to test patients before the onset of the COVID-19 pandemic, which would have allowed us to evaluate the specificity of this test in detecting an anti-Covid vaccine response. Nevertheless, the positivity rate at V1 in patients with NPI was only 6% for the ELISpot IFN-γ alone, but this rate increased to 52% when the four Th1 cytokines were included in the model. By comparison, the positivity rate at V1 for PI subjects was 62% when solely considering IFN-γ, and it reached 87% when at least one positive cytokine among the four Th1 cytokines included in the model was considered.

### Correlation between the various cytokines produced by CD4^+^T cells for vaccine response detection

**Figure 4:**
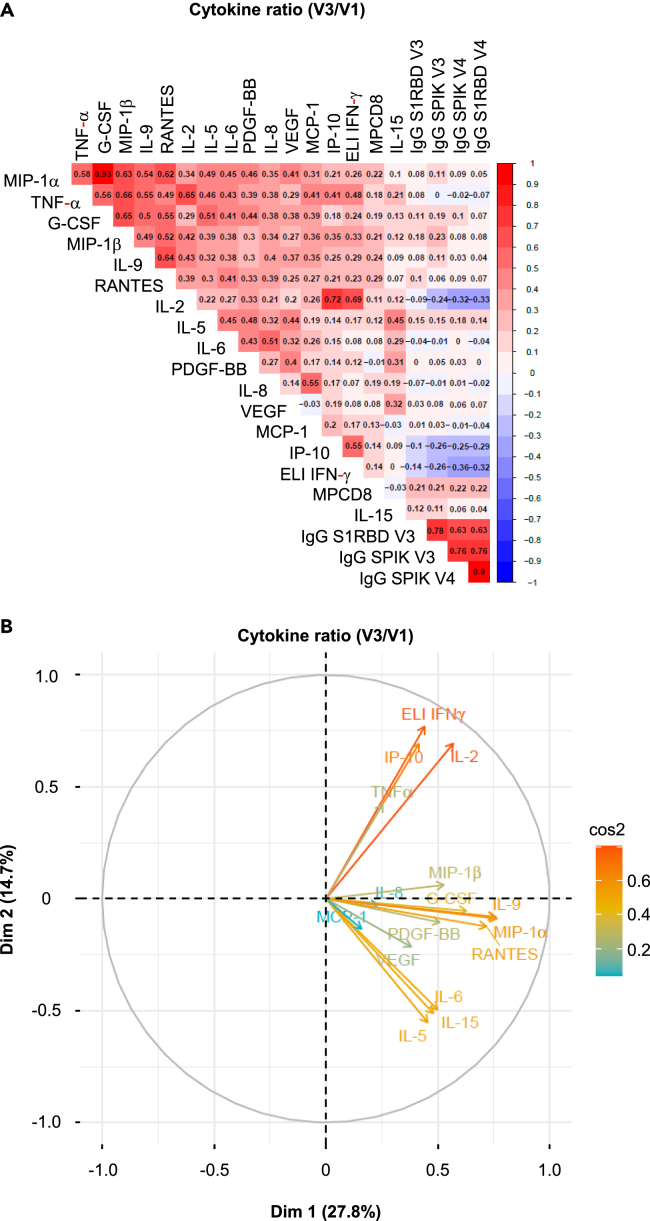
Correlation among various cytokines defining the vaccine response to CD4^+^T cell cellular and humoral responses (A) Non-parametric Spearman test for the analysis of the correlation matrix illustrates the strength of the correlation between each cytokine produced by CD4^+^T cells following the vaccine response, as defined by the V3/V1 ratio, and the serological response. The concentration of anti-spike or RBD IgG antibodies at 3 and 6 months is incorporated into this correlation matrix as a surrogate marker of serology. The scale for the correlation is shown on the right side of the matrix. Negative correlations are depicted in blue, while positive correlations are shown in red (*n* = 128). (B) Principal Component Analysis (PCA) biplots for the pattern of multicytokine secretion by CD4^+^T cells in response to the vaccine (V3/V1). In each biplot, the lengths of arrows correspond to the magnitude of the variable (approximating its variance). The angles between arrows (cosine) approximate their correlation. All arrows are labeled with the respective variables they represent.

A correlation matrix (Figure 4A) and a data projection using reduced dimensions, such as principal component analysis type (Figure 4B), showed a high degree of similarity among the four predominant cytokines (IFN-γ, IL-2, TNF-α, IP-10), which further emphasized the likelihood of a coordination response to the vaccine.

Additionally, a second group of cytokines also appeared to be correlated (FGF, MIP1-β, MIP1-α, IL-9, G-CSF, PDGF-β), as did a third group (IL-5, IL-6, IL-15, MCP1, IL-1-β) (Figure 4B). While, these groups of cytokines may not fit into conventional cytokines classification (such as Th2, Th17, Th22, and so forth), they do suggest a coordinated production of certain cytokines.

### Correlation between the cytokine produced by CD4^+^T cells and humoral response

We did not identify any correlation between the production of the 16 predominant cytokines produced by CD4^+^T lymphocytes and the humoral response, which was detected by the concentrations of different types of antibodies. These antibody concentrations were categorized using the median values for IgG S1 RBD V3 (D57), IgG Spike V3 and V4 (6 months after the first immunization), and IgG S1 RBD V4 in response to the vaccine (Figure 4A).

The same results were found when the absolute cytokine value at V1 or V3 was plotted against the serological values (Figure S6).

**Figure 5:**
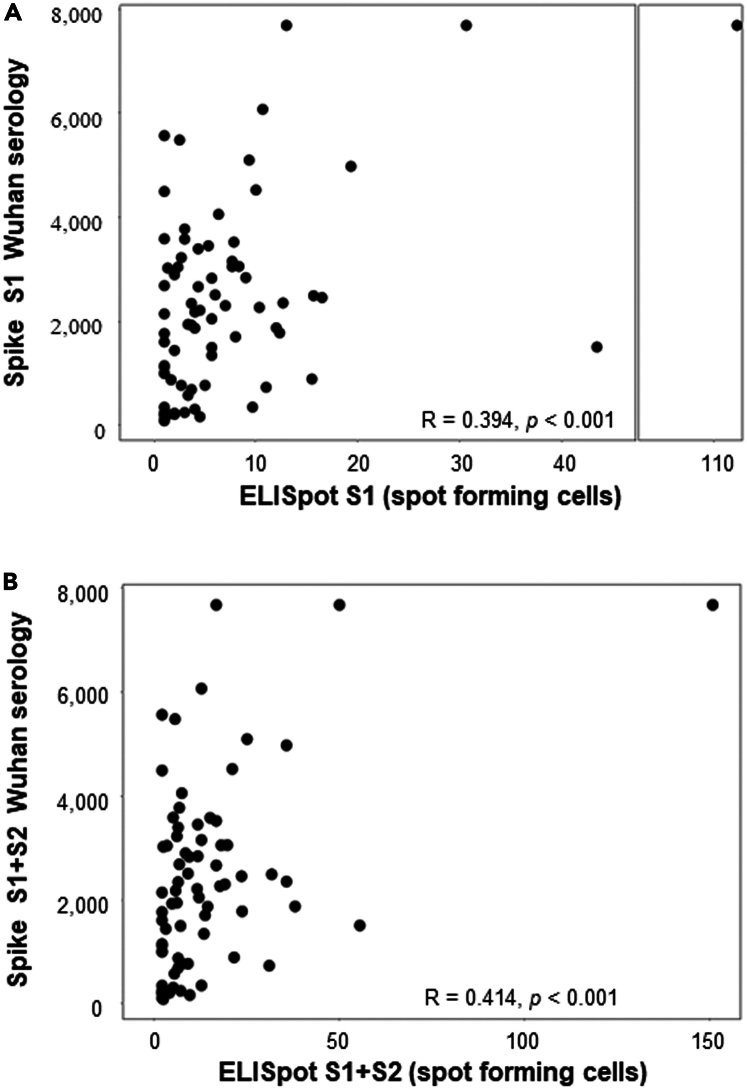
Correlation between IL-21 produced by anti-spike CD4^+^T cell and spike serology IL-21 ELISpot assay against S1 (A), or S1+S2 (B) peptide MP on sorted CD4^+^T cells was performed in a cohort of patients vaccinated with the BNT162b2 vaccine (*n* = 108). The ELISpot results are expressed as the number of spots/10^5^ cells. These results were correlated with spike serology performed at the same time point as the IL-21 ELISpot results.

Previous studies have shown that IL-21 is an essential cytokine produced by CD4^+^T cells to help B lymphocyte maturation.^29^ We assessed therefore whether an ELISpot IL-21 assay on CD4^+^T cells, conducted simultaneously at the same time as serology on samples obtained closest to the time of breakthrough infection, could predict the humoral response. A clear correlation between the intensity of IL-21, secreted by CD4^+^ T cells activated using a pool of peptides obtained from the S1 RBD domains or S2 of the Spike protein from the Wuhan strain, and the anti-Wuhan Spike (S1+S2) or S1 serological response as detected by Elisa (Figures 5A and 5B). Moreover, a trend emerged between the IL-21 secretion triggered by the S1 domain and the neutralization test targeting the same Spike domain from the Wuhan strain (r = 0.291 and *p* = 0.011).

### Specific cytokine profile derived from CD4^+^T cells predicts anti-Spike CD8^+^T cell response

**Figure 6:**
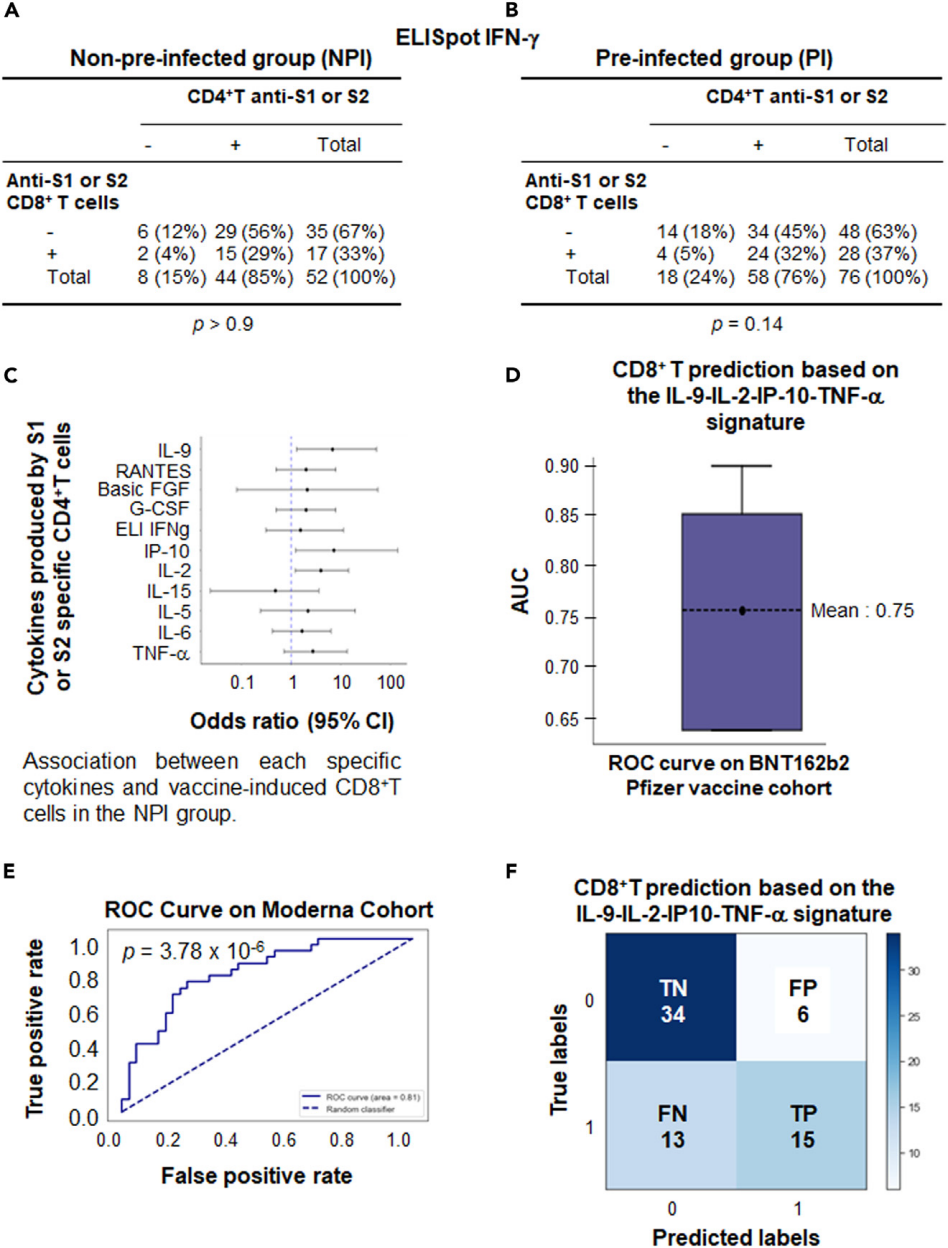
Profile of cytokines produced by CD4^+^T cells could predict the vaccine induced CD8^+^T cell response using a machine learning approach Correlation via Pearson’s Chi-Squared test was sought between the positivity of the ELISpot IFNγ performed on CD4^+^T cells against S1 or S2 after vaccination (D57) in non-pre-infected (*n* = 108) (A) or pre-infected (*n* = 104) (B) volunteers and the parallel induction of CD8^+^T cells against S1 or S2. (C) Correlation was sought between the positivity for the V3/V1 ratio criteria of each cytokine produced by CD4^+^T cells sensitized by S1 and S2 after vaccination (D57) in non-pre-infected volunteers and the parallel induction of CD8^+^T cells against S1 or S2 (detected by ELISpot IFNγ) using the statistical Cox test. (D) An algorithm was established using machine learning by training 80% of the Pfizer cohort with a Gradient Boosting algorithm (XGB). The model was validated on 20% of volunteers from the unused Pfizer cohort using 5-fold cross-validation to demonstrate the stability of the model. Boxplots representing the resulting AUC for the resulting models are shown. (E) ROC curve calculated on the mRNA-vaccinated Moderna cohort dataset (*n* = 68). The dashed diagonal line represents random classification. AUC and *p*-values are shown. (F) The confusion matrix generated by the model summarizes the model’s performance. The matrix’s diagonal elements represent the number of corrected predictions (True Negative [TN] and True Positive [TP]), while the off-diagonal elements represent incorrect predictions (False positive [FP] and False negative [FN]). The Fisher exact test was used to determine the *p* value.

Using the ELISpot IFN-γ method, the positive response of anti-spike CD4+T and CD8+T cells were not correlated on D57. This response was absent both in NPI volunteers (Figure 6A) and PI volunteers (Figure 6B) who were vaccinated with BNT162b2 mRNA. In case of NPI participants, a Luminex assay was performed on the supernatant of CD4^+^T cells sensitized with a pool of peptides derived from S1 and S2 domains. Initially, the cytokine production was considered as a qualitative statistical parameter. It was considered positive when the median fold change of the V3/V1 ratio in the population was ≥2. Notably, a significant correlation was observed between the induction of anti-spike CD8^+^T cells on D57 and the positivity of the V3/V1 ratio for IL-9 (*p* = 0.033) and IL-2 (*p* = 0.026; Figure 6C, logistic regression). A trend was also noted for other cytokines such as IP-10 (*p* = 0.068) and TNFα (*p* = 0.16; Figure 6C), although not for Th2 cytokines (IL-5 and IL-6; Figure 6C). Concerning pre-infected subjects, a correlation was exclusively found with IL-9 and Rantes (CCL5; Figure S7).

To further support this correlation, the four cytokines (IL-9, IL-2, IP-10, TNF-α) associated with the induction of CD8^+^T cell responses in patients with NPI were selected using a machine learning approach based on the XGB model for this purpose (See STAR methods). Additionally, along with the positivity or non-positivity of the V3/V1 ratio for each cytokine, the quantitative value of this ratio and the absolute value of cytokine concentrations at V3 were integrated into the model (Figure S8). For an algorithm development, the model was trained using 80% of the mRNA BNT162b2 vaccine Pfizer data (*n* = 54), while preserving the statistical distribution related to the induction of vaccine-specific CD8^+^T cells. The hyperparametrized model was then validated on the remaining 20% of the Pfizer cohort. Although the number of patients was low in this validated cohort (*n* = 24), the model showed an AUC of 0.75 for predicting the vaccine-induced CD8^+^T cells (Figure 6D). To confirm the value of this signature, the hyperparametrized model was tested on a similar cohort of NPI volunteers vaccinated with the mRNA-1273 Moderna vaccine. The ROC curve (Figure 6E) displayed an AUC of 0.81 (*p* = 3.78 × 10^−^^6^), providing a strong confirmation for the value of this original signature based on various parameters derived from these four cytokines (IL-9, IL-2, IP-10, TNF-α). The confusion matrix resulting from this model (Figure 6F) demonstrated a specificity of 85% for the test, with a positive predictive value of 71% and a sensitivity of 53%.

### Value of the four cytokines (IL-2, TNF-α, IL-9, and IP-10) produced by CD4^+^T cells to predict antigen specific CD8^+^T cells frequency in mice

**Figure 7:**
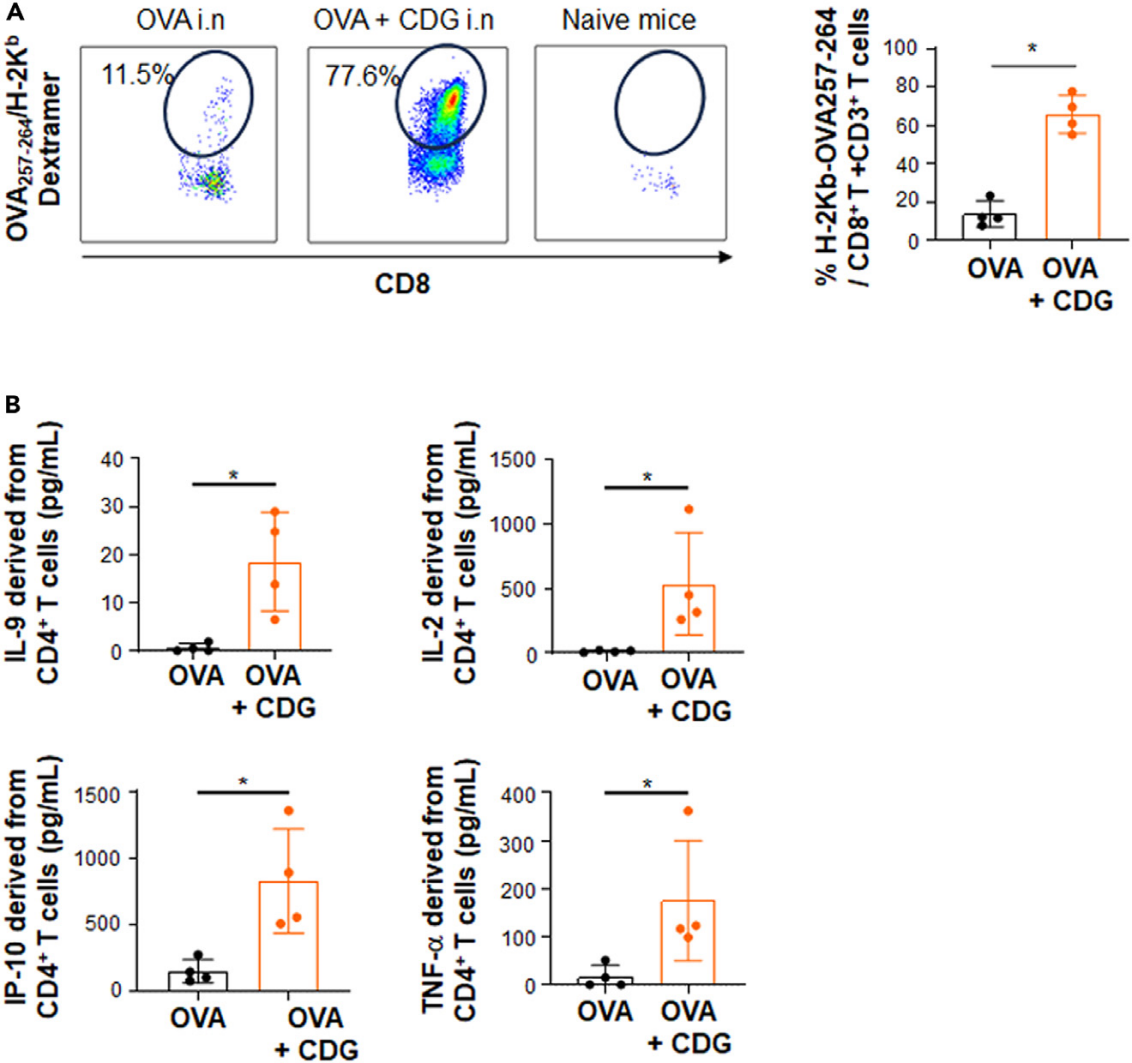
Value of the CD4^+^T cells derived cytokine signature in predicting CD8^+^T cell response in mice Mice (*n* = 4) were immunized by the nasal route with ovalbumin (OVA; 100 mg), either alone or combined with the adjuvant C-di-GMP (10 mg), on D0 and D14. On D21, BAL was recovered and the frequency of CD8^+^T cells was quantified using an OVA_257-264_ H-2K^b^ dextramer. After perfusion, lungs were harvested, and purified CD4^+^T cells (10^5^ cells) were plated in 96-well plates with splenocytes derived from naive mice that were either sensitized or not with the long OVA peptide (TEWTSSNVMEERKIKV [OVA_265–280]_). After 36 h, supernatants were collected and tested for the presence of IL-9, IL-2, TNFα, and IP-10. (A) Induction of anti-OVA_257-264_ CD8^+^T cells in mice vaccinated with OVA alone or in combination with the adjuvant C-di-GMP. (B) Cytokine concentrations in CD4^+^T cells supernatants derived from lung, sensitized with the long OVA peptide. The Mann Whitney statistical test was used for the analysis; Data are represented as mean ± SEM. ∗: *p* ≤ 0.05; ∗∗: *p* ≤ 0.01; ∗∗∗: *p* ≤ 0.001.

We then proceeded to assess whether the cytokine profile of CD4^+^T cells, based on the four cytokines selected and predictive of antigen-specific CD8^+^T cell response in humans, could be validated in mice using other vaccine platforms and routes of immunization. After administering mucosal (intranasal) immunization to mice with OVA, either as recombinant protein alone or in combination with a sting agonist adjuvant (C-di-GMP), we observed a significant induction of anti-OVA_257-264_ CD8^+^T cells within the bronchoalveolar lavage of mice. In the group that received OVA alone, approximately 13% of the CD8^+^T cells were positive for dextramer binding, a marker of antigen specificity. However, when the C-di-GMP adjuvant was added to OVA, this frequency increased to 73.95% (Figure 7A). Notably, no dextramer-positive CD8^+^T cells were detected in non-immunized naive mice (Figure 7A).

In addition, when comparing two groups of mice, a significant increase in the level of IL-9, IL-2, IP10, and TNF-α was observed in the group of mice with high concentrations of anti-OVA_257-264_ CD8^+^T cells. These mice had previously been immunized with a mixture of OVA and C-di-GMP, as opposed to the group immunized with OVA alone. The latter group showed significant but lower concentrations of dextramer^-^positive cells (Figure 7A). No detection of cytokines was observed in naive mice. A significant correlation between dextramer frequencies and the concentrations of the four cytokines (IL-9, IL-2, TNF-α, IP-10) was also found, as indicated by correlation coefficient ranging from 0.43 to 0.57, and a significant *p* value (<0.05) for all cytokines except TNF-α (*p* = 0.053; Figure S9A). However, these cytokines were mostly detectable at high frequencies of antigen-specific CD8^+^T cells. To address this aspect, we conducted subcutaneous immunization in mice using OVA vectorized by the B subunit of Shiga toxin. Previous work has shown that the efficacy of this vector in inducing CD8^+^T cells is sub-optimal in the absence of an adjuvant.^39^ In fact, the mean frequency of CD8^+^T cells induction was 0.6% (±0.3), which is quite low compared to the frequencies observed after mucosal immunization. In this context, IP-10 and IL-9 were no longer detected in the supernatants of CD4^+^T cell stimulated with the MHCII-restricted OVA peptide. Although TNFα and IL-2 were detected in one and three mice out of four, respectively, there was no significant correlation with the frequency of dextramer^-^positive CD8^+^T cells (Figure S9B).

## Discussion

In this study, we demonstrated that an algorithm based on the production of IL-2, TNFα, IP10, and IL-9 cytokines by CD4^+^T cells was able to predict subsequent CD8^+^T cell response following vaccination with BTN162b2. This predictive signature was not only validated in a second cohort of volunteers vaccinated with the mRNA-1273 vaccine (AUC: 0.81, *p* = 3.78.10^−^^6^), i.e., the same vaccine platform, but it also exhibited a specificity of 85% and a positive predictive value of 71%. Interestingly, in mice, the concentration of these same cytokines produced by CD4^+^ T cells correlated with the intensity of the CD8^+^T cell responses, which were elicited through mucosal immunization using a recombinant protein with or without an adjuvant. This implies that the identified algorithm could hold significance across different species, immunization routes, and vaccine platforms. Unlike the analysis in humans where the algorithm exclusively qualitatively predicted the CD8^+^T cell response only, it appears that in mice, the prediction is also linked to the intensity of the response. Nevertheless, the absolute cytokine induction values following vaccination were also integrated into the algorithm, as identified in the human context.

A study by Painter et al.^22^ had shown that initially induced Th1 CD4^+^T cells, defined by the marker CXCR3, were correlated with CD8^+^T cell concentrations measured after the booster immunization. However, this study did not define the Th1 cytokine profile of these CXCR3^+^CD4+T cells. This result could have been expected given that Th1 cells predominantly facilitate CD8^+^T cell response by the way of specific Th1 cytokines such as IFNγ, IL-2, and TNF-α, which are essential for the expansion and maturation of CD8^+^T lymphocytes.^40^ Typically, IFN-γ, a representative of the Th1 response, is the most frequently measured cytokine during vaccine immunomonitoring. Interestingly, our findings revealed no direct correlation between CD4^+^T cell response, as detected by an IFN-γ ELISpot assay, and subsequent CD8^+^T cell response. This prediction was influenced by other Th1 cytokines (IL-2, TNF-α, IP-10), alongside IL-9, produced by Th9 CD4^+^T cells. Notably, in tumor models, Th9 cells aid in promoting cytotoxic CD8^+^ T cells expansion and activation, through CCL20/CCR6-dependent recruitment of type 1 dendritic cells (DC1), specialized for the cross-presentation of antigen by MHC class I molecules to CD8^+^T cells.^41^^,^^42^^,^^43^ Furthermore, the use of IL-9 as an adjuvant in DNA vaccination has been reported to induce the development of IFN-γ-producing CD8^+^T cells.^44^ Additionally, regarding IP-10, it is important to note that this cytokine is also induced by IFNγ. However, in an exploratory study involving a wide array of cytokines including IFN-γ, IP10 emerged as the most reliable and sensitive test for detecting the T lymphocyte response to SARS-CoV-2.^45^

After administering the BTN162b2 vaccine, we observed an induction rate of CD8^+^T cells of up to 36.8% in vaccinated volunteers compared to 32.6% in those who were not previously infected. While these frequencies may appear lower than those reported in the literature,^22^^,^^46^ several parameters could account for these differences. Firstly, we measured CD8^+^T cells on D57 after the first vaccination, whereas it has been shown that both CD4^+^ and CD8^+^T cell responses contracted after D29 in participants vaccinated with the BNT162b2 vaccine,^47^ with CD8^+^T cells declining more rapidly than CD4^+^T cells.^48^ Secondly, the various techniques used to detect CD8^+^T cells do not have the same sensitivity and specificity. Using our ELISpot IFN-γ technique with a threshold of 10 spots/10^5^ cells, we observed only a basal pre-vaccine response in 3 out of 52 (5.7%) patients with NPI, confirming the high specificity of this test. This threshold, based on our previous laboratory studies and other reported in different context,^49^^,^^50^^,^^51^ has been set lower in other studies, which could contribute to certain discrepancies.^52^^,^^53^ Various studies have indicated that an ELISpot IFN-γ against the Spike protein is not able to detect cross-reactive memory T cells,^52^^,^^54^ in contrast to more sensitive assays such as a 7-day proliferation or 24-h activation-induced markers cytometry assay. Nevertheless, these latter tests remain less specific in discriminating response to SARS-Cov-2 from those to other seasonal coronaviruses.^17^^,^^55^ Our selected stringent ELISpot threshold might also explain the moderate sensitivity of our algorithm, around 53%, which could possibly miss low-frequency CD8^+^T cells. We have also shown in mice that this algorithm effectively predicts the induction of strong CD8^+^T cell responses but is less effective in detecting weak responses (<1% dextramer-positive cells among total CD8^+^T cells). Nevertheless, we have shown that the enrichment of the CD4^+^T lymphocyte population makes the test more sensitive than the direct sensitization of PBMCs (Figure S3). A rapid measurement of SARS-CoV-2 spike T cells in whole blood with direct cytokine measurement in the supernatant has also been reported but it did not discriminate CD4 and CD8^+^T cells.^56^

To eliminate possible confounding factors regarding the relationship between the CD4^+^T cells cytokine profile and CD8^+^T cells response, we examined patient age and initial disease severity in patients with PI. We found no correlation between CD4^+^T cell vaccine response measured by ELISpot IFN-γ or Luminex cytokine assay. Unlike vaccine-induced B cells and antibody responses, decreasing with age,^57^^,^^58^^,^^59^^,^^60^ other studies have reported no significant age-associated changes in the induction of antigen-specific T cell responses.^22^^,^^52^ In fact, the influence of age becomes more pronounced in patients over 80 years,^61^^,^^62^^,^^63^ a demographic that is less represented in our cohort. Similarly, we did not observe a clear difference in the vaccine-induced CD4^+^T cell responses that could discriminate between the PI group (with or without symptomatic COVID-19). This aligns with other studies that have shown no impact of previous infection severity on the T cell response,^52^^,^^64^ although contrasting results have also been reported.^54^ The variably in classifying symptomatic infections and their severity cross studies could account for these discrepancies.

T follicular helper cells are primarily required to help the B cell proliferation and production of high-affinity antibodies within the germinal center of secondary lymphoid organs.^29^ The secretion of IL-21 currently represents the strongest indicator of Tfh function in peripheral blood.^65^ In parallel to the predicted correlation between CD8^+^T cell induction and the cytokine profile of the CD4^+^T cell response, we showed a significant association between ELISpot IL-21 measurement and the serological response against both the S1 and S2 domain of the SARS-CoV-2 Spike protein. Moreover, a trend was also observed between ELISpot IL-21 and a neutralization test, specifically against the Spike Wuhan protein’s S1 domain (r = 0.291 and *p* = 0.011). Similarly to our findings, other research groups have shown that the frequency of Tfh defined by CXCR5, BCL6, and CD40L phenotypic markers (without including IL-21 production), correlates with the induction of a durable and neutralizing humoral response against SARS-CoV-2.^22^^,^^66^^,^^67^ The value of monitoring IL-21-producing Tfh cells via cytometry has been highlighted during the development of various vaccines, including those against HIV, H1N1 or H5N1, as this metric correlates with a durable and neutralizing humoral response.^65^^,^^68^^,^^69^ In contrast, our results, based on an ELISpot IL-21 assay, could simplify the detection of Tfh cells through a single technological platform, enabling the comprehensive profiling of CD4^+^T cell cytokines, while cytokine assays such as Luminex can be directly performed on cell supernatants. In this context, we preferred to collect supernatants from plates without coating antibodies to minimize the potential biases associated with cytokine trapping.

Furthermore, our study highlights the utility of selecting a CD4^+^T cell detection test based on a cytokine profile rather than relying solely on an ELISpot IFN-γ test. This shift can increase the sensitivity of the test for detecting a vaccine response. However, this increase in sensitivity is accompanied by an increase in positive test results during pre-vaccination sampling. The positive responses in NPI subjects could be explained by cross-reactions with seasonal coronaviruses already found using other techniques.^1^^,^^17^^,^^28^^,^^55^^,^^70^^,^^71^^,^^72^^,^^73^ This technique has also made it possible to characterize and compare the vaccine response between PI and uninfected volunteers. Before vaccination, PI individuals had an anti-SARS-CoV-2 CD4^+^T cell response characterized by a Th1 cytokine profile (IFN-γ, IL-2, IP-10, TNF-α). Considering that the mean duration between pre-infection and the first vaccination was 9 months, these results indicate a degree of persistence in the polyfunctional CD4^+^T cell response after infection. However, despite this, PI vaccinated individuals did not exhibit a greater quantity or variance in the V3/V1 ratio of cytokines produced by CD4^+^T cells, one month after the booster dose than NPI volunteers. These results are in line with previous research indicating that hybrid immunity is associated with increased antibody responses, but comparable magnitude of T cell responses.^24^^,^^74^^,^^75^

One of the limitations of this study is that our cohorts were not designed to directly correlate the impact of the observed cytokine CD4^+^T cell profile with protection against infection.

Overall, this study reveals that a CD4^+^T cell cytokine profile, combining a signature of four cytokines (IL-2, IP-10, TNFα, and IL-9) with an ELISpot IL-21 test, offers a comprehensive overview of the coordination between humoral and CD8^+^T cell responses post-vaccination. This immunomonitoring approach proves particularly valuable for predicting the CD8^+^T cell response. It is well-established that these cells play an important role in protecting against severe forms of Covid-19 and other viral infections, but also in certain clinical contexts such as cancer.^1^^,^^76^^,^^77^^,^^78^ However, unlike detecting CD4^+^T cells, where using 15-mer peptides overlapping the entire protein sequence of the interest is well adapted without the need for patient HLA typing, precise the identification of CD8^+^T cells is a cumbersome task, as optimal 8–10 mer peptides must cover diverse HLA class I alleles of individuals. Therefore, utilizing a CD4^+^T cell cytokine profile may indeed aid in predicting CD8^+^T cell responses in specific scenarios, such as emerging infectious diseases or the characterization of novel complex multi-antigenic vaccines or in immunocompromised patients with low cell count. In situations where the blood volume is not limited and the optimal peptides for CD8^+^T cells are available, this test for the detection of CD4^+^T lymphocytes with cytokine profile is obviously of less interest than outside a cognitive aspect to show the potential integration of the adaptive immune response.

### Limitations of the study

The correlation between the cytokine profile of the post-vaccination CD4^+^T cell response and the CD8^+^T cell response has only been demonstrated in patients who were not pre-infected prior to vaccination. In humans, the vaccines used were based solely on the mRNA.

Longitudinal follow-up is not available to assess whether this integration of the different immune components persists over time.

## STAR★Methods

### Key resources table

**Table:** 

REAGENT or RESOURCE	SOURCE	IDENTIFIER
**Antibodies**		

Anti-CD16-32	In vitrogen	16-0161-86
Anti Kb-OVA257-264 Dextramer PE	Immudex	JD02163 PE 150
Anti-CD3 percpCy5.5	eBioscience	45-0031-82
Anti-CD8 BUV 496	Becton-Dickinson	741127
Anti-human CD3 BV510	BioLegend	300448
Anti-human CD4 FITC	BioLegend	300506
Anti-human CD8 PE	BioLegend	301008
Anti-human CD4 BV510	BioLegend	300546
Anti-human CD14 FITC	BioLegend	325604
Anti-human CD19 FITC	BioLegend	302206
Anti-human CD8 BV421	BioLegend	344748
Anti-human CD86 PE	BD PharMingen	555658
Isotype IgG1 PE	BD PharMingen	555749
Anti-human HLA-DR APC	Beckman Coulter	IM3635
Isotype IgG1 APC	Beckman Coulter	IM2475
Zombie Nir viability dye	BioLegend	423106
FcR Blocking Reagent	Miltenyi Biotec	130-059-901

**Chemicals, peptides, and recombinant proteins**		

Peptides S1 and S2 Wuhan Strain	JPT	PM-WCPV-S-2
Peptide OVA265-280	Genosphere	
PMA	Sigma-Aldrich	P8139
Ionomycin	Sigma-Aldrich	I0634
Ovalbumin	Sigma	A5503
Ketamine	Virbac	QN01AX03
ROMPUN 2% Xylazine	Elanco	Not found
RPMI 1640 Medium	Gibco	72400–021
Heat inactivated fetal calf serum	Biowest	S140B-500
Sodium Pyruvate	Gibco	11360–070
Non-essential amino acids	Gibco	11140–035
Penicillin and streptomycin	Gibco	15140–122
2-β mercaptoethanol	Gibco	31350–010
Phosphate Buffer saled	Gibco	10010–015
EDTA UltraPure	Gibco	15575–038
Live/dead Fixable aqua dead cell stain	In Vitrogen	L34957
C-di-GMP	InvivoGen	vac-nacdg
Collagenase IV	Gibco	17104–019
DNAse I	Roche	04 536 282 001
C.T.L test medium	CTL Immunospot	CTLT-005
GlutaMAX Supplement	GIBCO	35050038
PBS 1X	GIBCO	10010056
Bovine Serum Albumin solution	Merck	A1595
EDTA solution	Merck	E7889
Cell Staining Buffer	BioLegend	420201

**Critical commercial assays**		

Elispot IL-21	Mabtech	3540-4APW-10
Elispot IFNg	Diaclone	856.051.020P
Bioplex Prohuman cytokine 27-Plex Pane	Biorad	M500KCAF0Y
V-Plex coronavirus panel 2 for IgG(CK153690)	Meso Scale Discovery	K15383U-2
Multiplex Immunoassay CXCL10	BioRad	12002244
LEGENDplex™IL-9-IL-2-TNFa	Biolegend	741044
EasySep™ Mouse CD4 Positive Selection Kit II	STEMCELL	18952
Human CD4 MicroBeads	Miltenyi Biotec	130-045-101
LD separation columns	Miltenyi Biotec	130-042-901
Pre-Separation Filters, 30 μm	Miltenyi Biotec	130-041-407

**Experimental models: Organisms/strains**		

C57BL/6 mice	Janvier	

**Software and algorithms**		

Bio-plex Manager software	Biorad	Standard Edition
Legend Plex data analyzer software	Biolegend	Version 8
FlowJo	Becton Dickinson	Version 10.10
Immunospot SC	C.T.L Immunospot	Version 7.0.26.0
Kaluza Analysis	Beckman Coulter	Version 2.1

**Other**		

C.T.L Reader S6 Ultimate	C.T.L Immunospot	Not Found
Navios 10 colors	Beckman Coulter	BB8782
Bioplex 200 Instrument	Bio-Rad	171000201
Quickplex SQ120	Meso scale Discovery	Serial Number 1300170428908
Gentle Macs Dissociator	Miltenyi	130-093-235
BD LSRFortessa X20	Becton Dickinson	657675R1

### Resource availability

#### Lead contact

Further information and requests for resources and reagents should be directed to Eric Tartour (eric.tartour@aphp.fr) and will be fulfilled by the lead contact.

#### Materials availability

This study did not generate new unique reagents.

#### Data and code availability

This paper does not report original code. Any additional information required to reanalyze the data reported in this paper is available from the lead contact.

### Experimental model and study participant details

#### Cohorts of vaccinated volunteers

##### Pfizer and Moderna cohorts

In APHP CoviCompareM (ClinicalTrials.gov NCT04748471) and ANRS002S CoviCompareP (ClinicalTrials.gov NCT04824638) trials, adults not previously infected with SARS CoV-2 (negative SARS-CoV-2 serology and PCR at inclusion and no previous history of COVID-19), received two full doses of either mRNA-1273 (Moderna) or BNT162b2 (Pfizer) vaccine, 28 days apart. In CovicompareP trial, were also included participants with a documented history of SARS-CoV-2 infection at least 5 months before inclusion. They were categorized into three subgroups according to the severity of their previous infection i) asymptomatic ii) moderate COVID-19 infection, defined as symptomatic but not hospitalized, or hospitalized without oxygen requirement, and iii) severe infection defined as requiring hospitalization and oxygen supplementation. These pre-infected patients received a single dose of the BNT162b2 vaccine according to the French COVID-19 immunization guidelines of that time. The participants were healthy adults or those with stable medical condition, defined as disease not requiring changes in therapy or hospitalization for worsening disease within 3 months before enrollment, or expected significant changes in the foreseeable future. In these two cohorts, patients across three age groups: 18–45 years, 65–74 years and >75 years, were enrolled. For ANRS002S CoviCompareM, the analysis involved a random sample of 68 participants (39 men and 29 women) equally distributed among the three age groups. For ANRS002S CoviCompareP, the analysis was performed on a sample of 128 participants distributed across age groups (Table S1) in both the non-preinfected group (NPI [*n* = 52 (25 men and 27 women)]) and in the previously infected group (PI [*n* = 76 (42 men and 34 women)]; Table S1). Evaluation was conducted at two time points: D0 (before vaccination, V1) and D57 (one month after the vaccine boost, V3).

The 2 clinical protocols and all amendments had the approval of the Ethics Committee (CPP of Ile de France 1) and the national drug regulatory authority (ANSM). Signed informed consent was obtained from each participant. All methods were performed in accordance with the relevant guidelines and regulations.These cohorts have already been described.^79^^,^^80^

##### Breakthrough infection cohort

For ELISpot IL-21 analysis, we examined 108 evaluable participants from an ancillary CovicompareP study involving cases of breakthrough infections that occurred during the period of Omicron variant circulation. Each individual’s samples consisted of their last available pre-infection sample of biologically confirmed breakthrough infection cases and non-infected controls. These samples were matched in a 1:1 ratio, considering factors such as previous infection status, age group, and the time of sample collection.

#### Vaccination model in mice

Female C57BL/6J mice, aged 6 to 8 weeks, were purchased from Janvier Labs and bred under specific pathogen-free conditions in our animal facility. The sex of the mice was selected because we have already protocols using female. Mice were divided into groups of not vaccinated (naive) mice and those subjected to intranasal (i.n; dropwise) or subcutaneous (s.c; left flank) vaccination at D0 (Prime dose) and D14 (Boost dose). These vaccination involved the administration of Ovalbumin (OVA, 100 μg) either alone or in combination with the adjuvant C-di-GMP (10 μg, InvivoGen), or with OVA vectorized using the B subunit of Shiga toxin (STxB-OVA, 20 μg) without adjuvant, following a previously described protocol.^81^ On D21, after intraperitoneal anesthesia with ketamine (10 mg/kg) and xylazine (80 mg/kg), bronchoalveolar lavage (BAL) was performed, and lung (for i.n route) and spleen (for s.c route) samples were collected. Subsequently, immune responses were assessed for these samples. All protocols were approved by French Ethical Committee (Apafis #29315).

### Method details

#### Peptides

Megapool of peptides S1 and S2 derived from the corresponding proteins include 157 and 158 15-mer overlapping peptides from S1 and S2 proteins respectively from the original Wuhan Strain. They were used for anti-spike CD4^+^T cell detection and were purchased at JPT peptide technologies (Berlin, Germany).

MPCD8E is a mega pool of experimentally defined 8–9 amino acid peptides (*n* = 454) derived from the Wuhan Spike of SARS-Cov-2. These peptides were identified^82^ and were provided to our laboratory by the research group led by A Sette (La Jolla, USA).

#### ELISpot IFN-γ to detect anti-spike T cells

The cellular immune response was assessed *in vitro* by measuring IFN-γ production from spike-specific CD4^+^T and CD8^+^T cells using the ELISpot technique (Diaclone, Besançon). Samples were collected at baseline (D0 = Visit1 [V1]) and one month after the vaccine boost for NPI or 2 months after the first dose for PI (V3). CD4^+^T cells were obtained from thawed peripheral blood mononuclear cell through positive selection with a system MACS cell Separation using beads CD4 and LD columns (Miltenyi Biotec, Paris). These cells were then sensitized for 20 h at 37°C with a pool of 15-mer overlapping peptides (*n* = 315) derived from the S1 or S2 protein of the wild-type SARS-CoV-2 Wuhan strain (1 μg/mL, JPT peptide technologies, Berlin, Germany). Negatively selected CD8^+^T cells were sensitized with the mega pool CD8 (MPCD8)^82^ for 20 h at 37°C. Each subject had a negative control (unstimulated cells in CTL-test medium, Bonn, Germany) and a positive control (cells stimulated with 100 ng/mL PMA 1 μM ionomycin (Sigma Saint-Quentin-Fallavier, France) for quality control. Post-incubation, plates were revealed according to the manufacturer’s instructions, then scanned and analyzed on a C.T.L reader (S6 Ultimate). A response was considered positive if the number of spots in wells stimulated with spike specific peptides was at least 2-fold greater than the number of spots in the negative control, using a cutoff of 10 SFC/10^5^ cells after background subtraction as previously described.^49^ A positive response to the vaccine was defined as a 2-fold increase in cytokine-producing spike-specific CD4^+^T or CD8^+^T cells at one month after the second vaccination for NPI or 2 months after 1 dose of vaccine for PI compared to baseline (day 0 [D0]). For CD4^+^T cells, we considered a test positive if the T cells response against the pool of S1 peptides or S2 peptides followed the above criteria of positivity. CD8^+^T cells were sensitized with a pool of optimal 8–10 mer peptides derived from the spike.

#### ELISpot IL-21 test

The cellular immune response was assessed *in vitro* using samples obtained at the nearest time point before Covid infection in the breakthrough cohort. CD4^+^T cells were sorted and purified as previously described for ELISpot IFN-γ. Subsequently, the production of IL-21 from CD4^+^T cells through ELISpot (Mabtech AB, Sweden) was measured, after 48 h of stimulation with a pool of 15-mer overlapping peptides derived from the spike of Wuhan Sars-CoV-2 strain (1 μg/mL/peptide), following the same procedures as for ELISpot IFN-γ except that as this Elispot assay was compared to the spike (S1 and S2) and S1 serology, we provided the response to the S1 peptides pools separately. For each subject, a negative control (unstimulated cells in CTL-test medium) and a positive control (cells stimulated with 10 μg/mL PHA) were included. After an incubation period, plates were revealed according to the manufacturer’s instructions, then scanned and analyzed using a CTL reader (S6 Ultimate). A positive response was defined in the same manner as previously described for ELISpot IFN-γ.

#### Luminex assay

Cytokine production by CD4^+^T cells was assessed using Luminex technology with a Bio-Plex Pro Human cytokine 27-plex Panel (Bio-Rad, Marnes-la-Coquette, France) after 48 h of stimulation with a pool of 15-mer overlapping peptides derived from the Wuhan Sars-CoV-2 strain (1 μg/mL). CD4^+^T cells were obtained from samples collected at D0 and one month after the vaccine boost (V3), following the same procedures as previously described for ELISpot IFN-γ. For each subject, a negative control (unstimulated cells in CTL-test medium) and a positive control PMA-ionomycin (Sigma Saint-Quentin-Fallavier, France) were included. After 48 h of stimulation, supernatants of CD4^+^T cells cultures were harvested and then frozen at −80°C until assessment. The assay was performed according to the manufacturer’s instructions then acquired on a Bioplex-200 instrument (Bio-Rad, Marnes-la-Coquette, France). The analyte concentration was calculated using a standard curve with Bio-Plex manager software (Bio-Rad).

A vaccine response is considered positive if the cytokine concentration after sensitization with the specific peptides derived from a spike is >10 pg/mL (after subtraction background with medium) and the ratio of V3/V1 is greater than 2. The chosen positivity threshold of 2 is arbitrary, yet it aligns with what is frequently reported in the literature as the fold-change positivity threshold for the Elispot, from which our technique is derived.^83^^,^^84^^,^^85^ Additionally, this threshold of 2 has been consistently applied in many publications from our team over the past years.^49^^,^^50^^,^^86^

For the luminex assay, the cells were directly sensitized with the mixed megapool of S1 and S2 peptides.

#### Serology assessment

Antibodies against SARS-COV-2 were quantified in plasma using the MSD’s V-PLEX Coronavirus Panel 2 for IgG (K15369U), according to manufacturer’s recommendations. SARS-CoV-2 Panel 2 kits allowed the multiplex quantification of Spike (S), S1 receptor binding domain (RBD) in 96-wells plate. Briefly, after saturation of plates, diluted samples (1:1000, 1:30000 or 1:90000 according to antigens and periods), calibrators and controls were added to the plate and bound antibodies were then labeled with SULFO-TAG Anti-human IgG Antibody. Plates were read using MSD QuickPlex SQ120. Raw data was processed using MSD’s Discovery Workbench version 4.0. For quantitation of antibody responses, an 8-point calibration curve was run in duplicate on all plates. IgG concentrations were expressed in BAU/mL that were defined relative to the assigned values of the reference standard provided by MSD.

##### CD4^+^ T cells isolation from spleen and lung samples

Lungs were perfused with 2 mM PBS-EDTA and digested in RPMI-1640 medium supplemented with collagenase D (1 mg/mL, Sigma-Aldrich) and DNase I (30 μg/mL, Sigma-Aldrich). GentleMACS lung digestion programs 1 and 2 (Miltenyi Biotec) were used for dissociation, before and after 30 min incubation at 37°C, respectively. The resulting single-cell suspensions were filtered and washed. CD4^+^T cells were isolated from lung samples (i.n route) and spleen samples (s.c route) using EasySep Mouse CD4^+^ Selection Kit II (Stem cell). One hundred thousand splenocytes recovered from naive and presensitized mice with an IA^b^ restricted OVA-derived peptide (OVA_265–280_, TEWTSSNVMEERKIKV) for 2 h before coculturing them with 1 × 10^5^ (lung) or 2 × 10^5^ (spleen) CD4^+^T cells. Supernatants were recovered after 36 h of coculture at 37°C in 5% CO2, in RPMI-1640 medium (Gibco) supplemented with 10% heat-inactivated fetal calf serum (Gibco), 1% sodium pyruvate (Gibco), 1% non-essential amino acids (Gibco, Marnes-la-Coquette, France), 1% penicillin and streptomycin (Gibco), and 0.5 mM 2-β mercaptoethanol (Invitrogen). Cytokines secretion was quantified by Multiplex immunoassays for CXCL10/IP-10 (Bio-Rad) or IL-9, IL-2, TNFα (Biolegend) according to the manufacturer’s protocol. Data acquisition was performed on a Bioplex-200 instrument (Bio-Rad, Marnes-la-Coquette, France, for Bio-Rad reagents) or BD Fortessa X20 (BD Biosciences, for Biolegend reagents) and analyzed using a standard curve with Bio-Plex manager software (Bio-Rad) and Legendplex Data Analysis software (BioLegend).

##### Flow cytometry analysis in bronchoalveolar lavage and spleen

Bronchoalveolar lavage (i.n route) was obtained by the lungs flushing with 2 mM phosphate-buffered saline-EDTA (5 washes with 1 mL) through a cannula inserted into the trachea. Spleens (s.c route) were recovered and dissociated in RPMI medium. Labeling of anti K^b^ restricted OVA_257-264_ (SIINFEKL) specific CD8^+^T cells was initiated after Fc receptor blocking (anti-CD16/32, Invitrogen). Cells were stained with K^b^-OVA_257-264_ dextramer (Immudex) at room temperature for 20 min. After washing with PBS, cells were incubated for 20 min at 4°C with LIVE/DEAD Fixable Aqua Dead Cell Stain (Invitrogen), CD3 PercpCy5.5 (eBioscience), and CD8 BUV496 (Becton Dickinson (BD) Biosciences). Data were acquired using BD Fortessa X20 (BD Biosciences) and analyzed with FlowJo Software (BD).

#### Human flow cytometry

Before or after cell magnetic sorting, cells were first incubated 30 min at 4°C with a viability dye Zombie Nir (BioLegend) then washed twice with staining buffer (BioLegend). After this step, cells were stained 30 min at 4°C with cytometry antibodies: CD3 BV510, CD4 FITC and CD8 PE (BioLegend) to measure purity of cell enrichment or with CD4 BV510 (BioLegend), CD8 BV421 (BioLegend), CD86 PE (BD PharMingen), HLA-DR APC (Beckman Coulter) and CD14 FITC or CD19 FITC (BioLegend) to assess the persistence of antigen-presenting cells within CD4^+^ and CD8^+^ enrichments fractions. For monocyte analysis, FcR blocking reagent was added 10 min at room temperature before antibodies staining. After 2 washes, data were acquired using Navios 10 colors (Beckman Coulter) and analyzed with Kaluza (Beckman Coulter). All the reagents are also detailes in the key resources table.

### Quantification and statistical analysis

Categorical data were presented as frequency counts and percentages, while continuous variables were presented as median and interquartile range (IQR). The Wilcoxon test with a false discovery rate (FDR) correction was used to compare the cytokines V3/V1 ratio between the NPI and PI groups. Spearman correlation analysis was used to evaluate the correlation between cytokines and serological markers. Additionally, a principal component analysis was conducted to determine cytokine groups, with the first principal component determining the ordering of the correlation matrix. Logistic regression was used to study the association between induction of anti-spike CD8^+^T and the cytokines V3/V1 ratios. Statistical analysis was performed with R version 4.1.1.

#### Choice and selection of machine learning model (ML)

Multiple Linear Regression (MLR) with its penalty-augmented alternatives (ElasticNet), Random Forest, Support Vector Classifier, and Gradient Boosting (XGB, CAT), built under the Python framework, were tested for this task. The XGB was built using the XGBoost package,^87^ CAT was constructed using the CatBoost^88^ package, while the other models were built using the Scikit-learn library. Prior to training, a data preprocessing step standardized the data, centering values around the mean with a unit standard deviation using the built-in standard scalar package in the Scikit-learn library. Machine learning models were tuned using the RandomizedSearchCV in Scikit-learn, with optimization based on the “ROC-AUC” error metric.

##### Data splitting strategy

Regarding data splitting, a stratified cross-validation procedure was adopted for training and assessing ML models. This strategy involved a typical train-validation-test model validation approach. The Pfizer dataset was used as both training and validation dataset, following a k-fold cross-validation for model development. The Moderna dataset was then used to validate the predictive power of the resulting model.

Each ML model underwent a 5-fold stratified cross-validation process to assess its performance. Each step consisted in randomly holding back 20% of data for evaluation using the StratifiedKFold method from the Scikit-learn library^89^ in Python, while the remaining 80% was used for hyperparameter optimization. For each algorithm, a grid search with selected hyperparameters along with their respective ranges, was first constructed. Subsequently, a random grid search method from the Scikit-learn library was used. At the end of this step, the XGB machine learning model was selected.

The Moderna cohort dataset was employed to test the performance of the hyperparametrized model.

##### ML model explanation

SHapley Additive exPlanations (SHAP) analysis was conducted on the XGBoost model. The effect of the input features on predictions was assessed using the KernelExplainer package from the SHapley Additive exPlanations library^90^ in Python.

##### ML model evaluation

The predictive performance value of all trained ML models was assessed. This was achieved by determining the mean ROC-AUC through 5-folds cross-validation on the Pfizer dataset and subsequently on the Moderna test dataset after hyperparametrization. The ROC curve allowed defining a range of sensitivity and specificity of the classification as follows: sensitivity = TP/(TP + FN); specificity = TN/(TN + FP), where TP, TN, FP and FN represent the components of the confusion matrix: true positive, true negative, false positive and false negative, respectively. Additionally, the accuracy = (TN + TP)/(TP + FN + TN + FP) was determined.
