# Supplementary material for: Cytokine profile of anti-spike CD4+T cells predicts humoral and CD8+T cell responses after anti-SARS-CoV-2 mRNA vaccination
Source: iScience. 2024 Jul 2;27(8):110441. doi: 10.1016/j.isci.2024.110441 (PMC11298648; doi:10.1016/j.isci.2024.110441)
Supplement: Document S1. Figures S1–S9, Tables S1, and S2 [file mmc1.pdf]

iScience, Volume *27*

Supplemental information

Cytokine profile of anti-spike $CD4^{+}T$ cells predicts humoral and $CD8^{+}T$ cell responses after anti-SARS-CoV-2 mRNA vaccination

Nadine Benhamouda, Anissa Besbes, Rebecca Bauer, Nesrine Mabrouk, Gauthier Gadouas, Corinne Desaint, Lucie Chevrier, Maeva Lefebvre, Anne Radenne, Marie Roelens, Béatrice Parfait, Daniela Weiskopf, Alessandro Sette, Nadège Gruel, Marie Courbebaisse, Victor Appay, Stephane Paul, Guy Gorochov, Jacques Ropers, Said Lebbah, Jean-Daniel Lelievre, Ludger Johannes, Jonathan Ulmer, David Lebeaux, Gerard Friedlander, Xavier De Lamballerie, Patrice Ravel, Marie Paule Kieny, Fréderic Batteux, Christine Durier, Odile Launay, and Eric Tartour

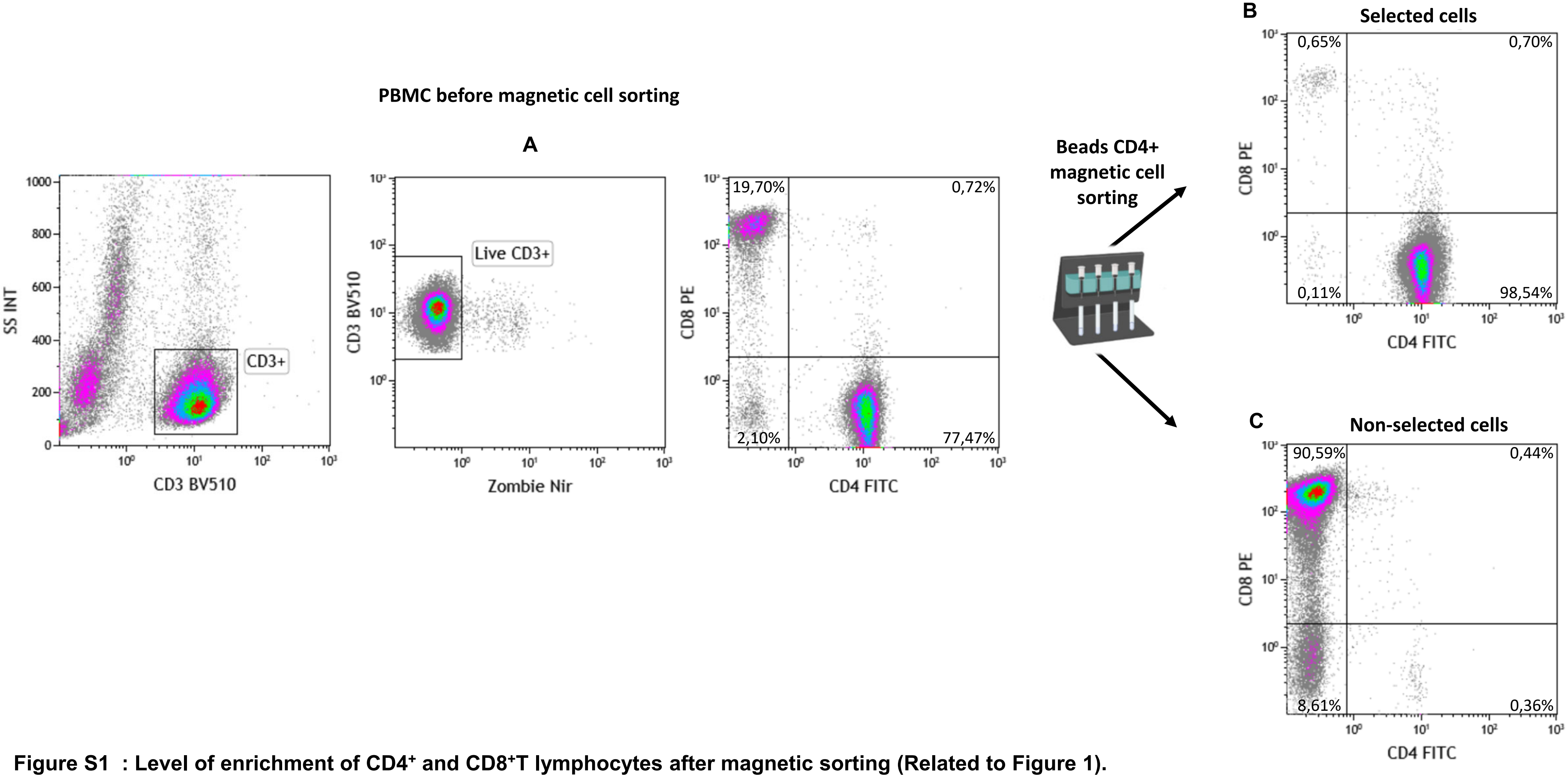

Figure S1 : Level of enrichment of $CD4^{+}$ and $CD8^{+}$T lymphocytes after magnetic sorting (Related to Figure 1).

(A) Percentage of gated T-CD4 and CD8 lymphocytes on live ($CD3^{+}$) T lymphocytes on PBMC (n = 4) before CD4 magnetic cell sorting

(B) After CD4 magnetic cell sorting: pourcentage of $CD4^{+}$T cells in gated CD3+T cells after elution of the cells binding in the $CD4^{+}$beads

(C) Percentage of $CD8^{+}$T cells in the eluted fraction of the non-binding cells to CD4 beads.

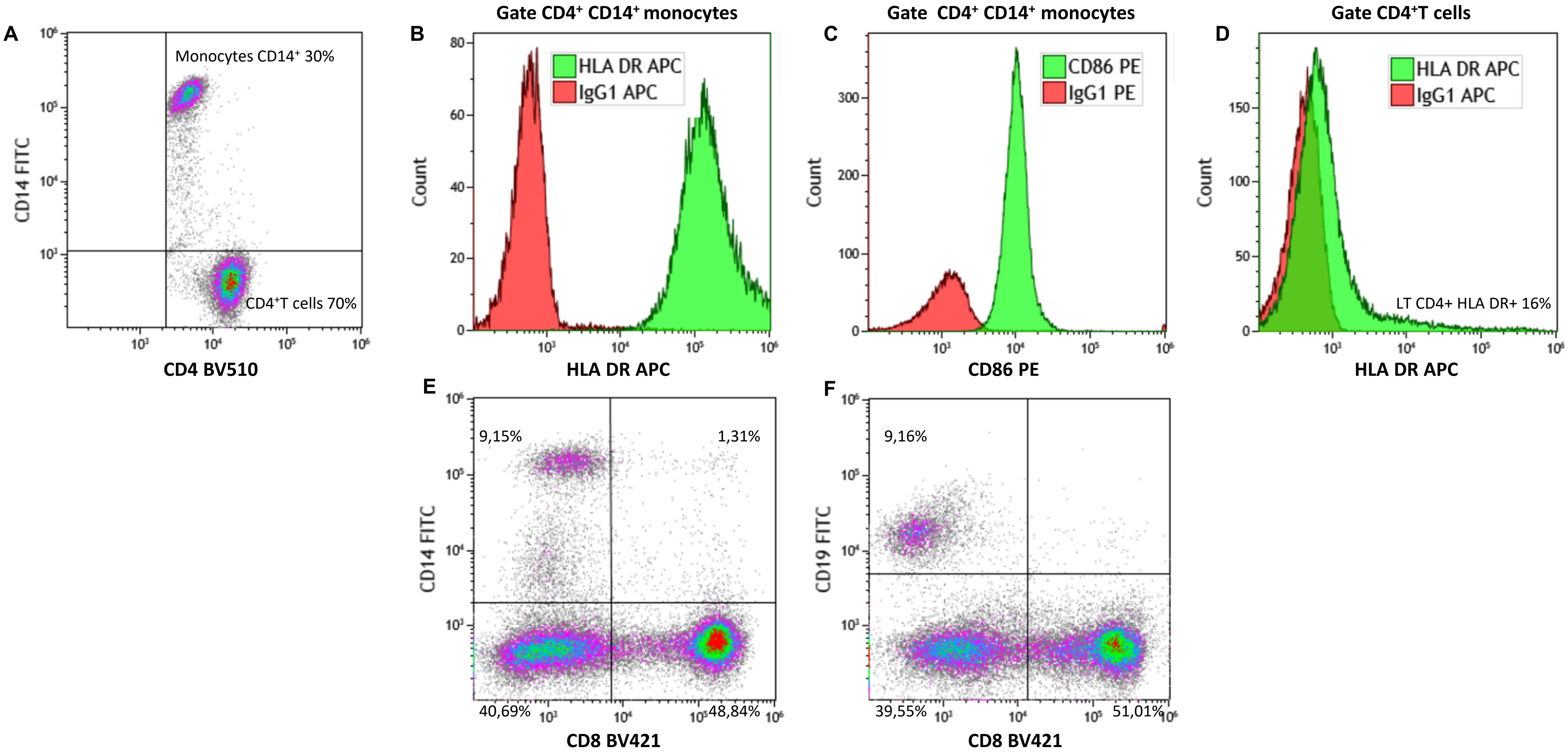

Figure S2 : Persistence of antigen presenting cells after CD4 and CD8 magnetic cell sorting (Related to Figure 1)

(A) Persistence of $CD4^+CD14^+$ monocytes in the samples (n = 4) after CD4 magnetic cell sorting,.

(B) Expression of HLA-DR by these monocytes ($CD4^+CD14^+$).

(C) Expression of CD86 by these monocytes ($CD4^+CD14^+$).

(D) A fraction of $CD4^+$T cells also express HLA-DR.

(E) After elution of the non-selected cells by the CD4 magnetic column, the enriched $CD8^+$T cells are also associated with other antigen presenting cells ($CD14^+$ monocytes

(F) Enriched CD8+T cells are also associated with $CD19^+$ B cells

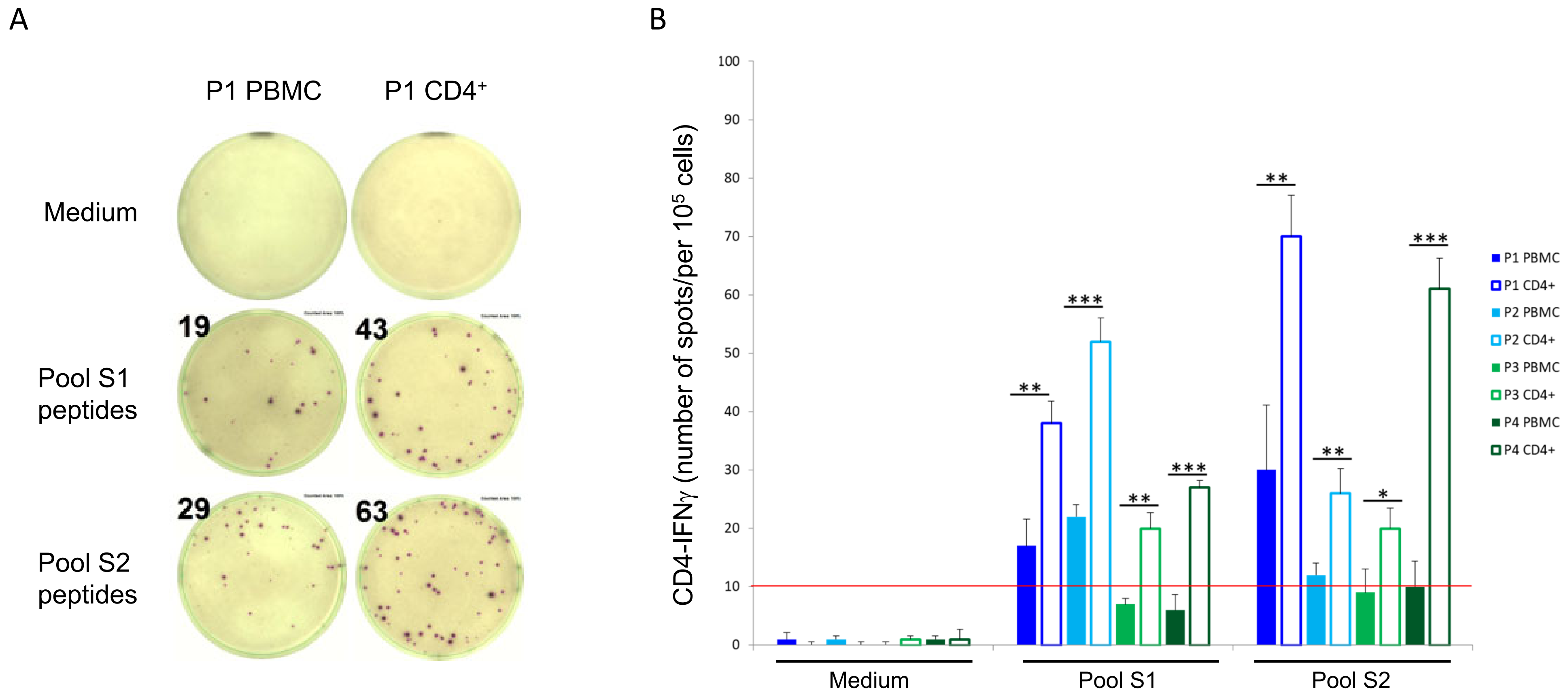

Figure S3 : Higher sensitivity for the detection of specific CD4$^+$ T cell after CD4$^+$T cell enrichment compared to the use of PBMC as antigen presenting cells (Related to Figure 1)

(A) Image of IFNγ Elispot wells scanned and counted for one patient after BNT162b2 mRNA vaccination (P1 = patient 1) before magnetic cell sorting (PBMC) and after magnetic cell sorting (CD4$^+$) and stimulated with a pool of 15-mer peptides S1 or S2 derived from the spike protein of SARS-Cov2 or unstimulated (medium) as negative control.

(B) CD4$^+$T cell response detected by IFNγ Elispot for 4 patients (P1 to P4) vaccinated with BNT162b2 mRNA under three conditions (medium, pool of peptides derived from the domain S1 or S2 of the spike protein) before magnetic cell sorting (PBMC) and after CD4$^+$ magnetic cell sorting. Filled histograms: patient before cell sorting (PBMC), empty histograms: patient after CD4$^+$ magnetic cell sorting (CD4$^+$); horizontal red line: positive threshold set at 10 spots for 10^5 cells per well after background subtraction. * $p < 0.05$, ** $p < 0.01$; *** $p < 0.001$ using unpaired t-test.

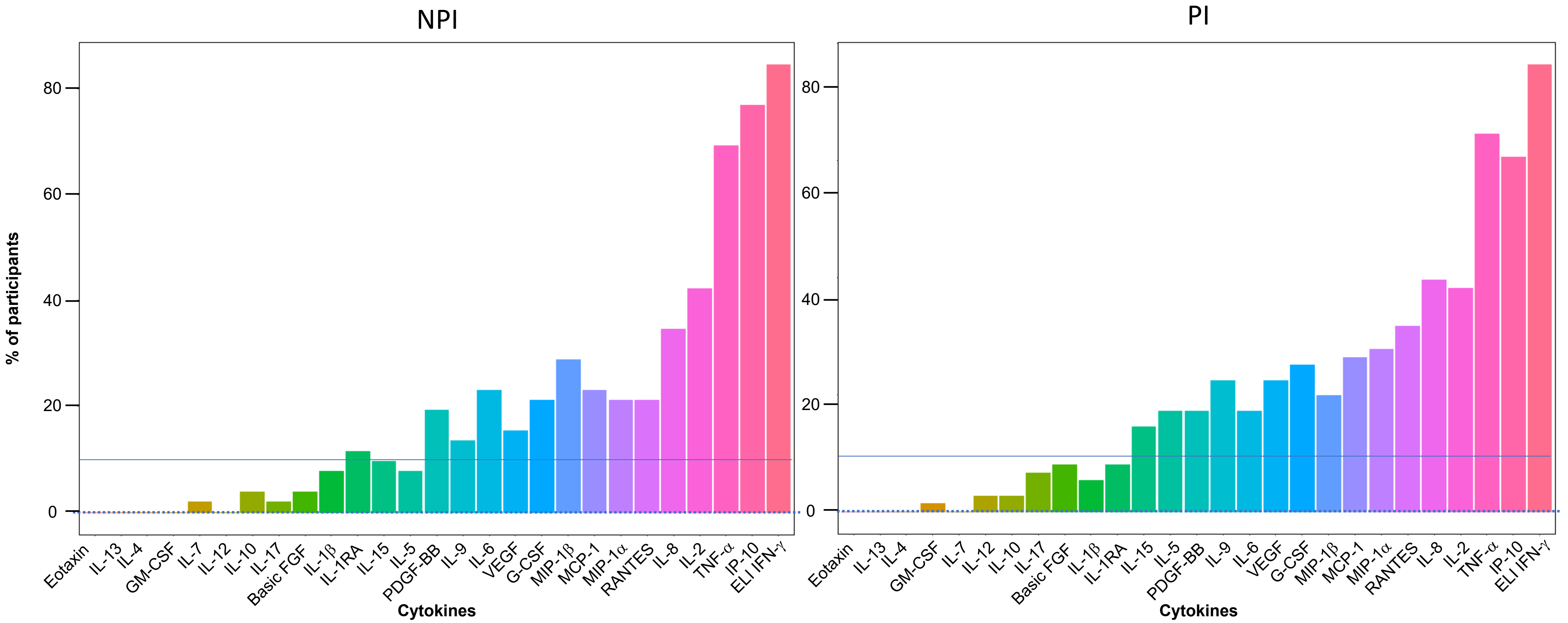

Figure S4: Percentage of vaccine-generated anti-spike CD4⁺T cell response depending on the cytokine measured in non-preinfected and infected patients (Related to Figure 1).

A total of 128 patients, including 76 who were pre-infected (Right) and 52 who were uninfected (Left), received vaccinations at V1 (D0) and V2 (D29). Only non-pre-infected volunteers received BNT162b2 vaccine (30 μg) at V1 and V2 and pre-infected volunteers received only the vaccine at V1. At V3, i.e., which is one month after the second vaccination for NPI and 2 months after the first vaccination for PI, the patients' $CD4^+$T cells were sorted and sensitized *in vitro* with a megapool of overlapping peptides covering the S1 protein and another pool for the S2 protein. An ELIspot (ELI) IFN-γ assay and a 27-cytokine Luminex assay, were then performed after 24 or 48 hours of incubation, respectively. The Luminex assay was used with supernatants of ELIspot IFNγ not coated with anti-IFNγ antibodies. The frequency of vaccine response for each cytokine, as determined by the V3/V1 ratio $\geq$ 2, and a concentration of the cytokine $\geq$ 10 pg/ml (after background subtraction when cells were sensitized with medium) is shown. The threshold for vaccine response detection for a given cytokine is indicated by the dotted line at 10% frequency

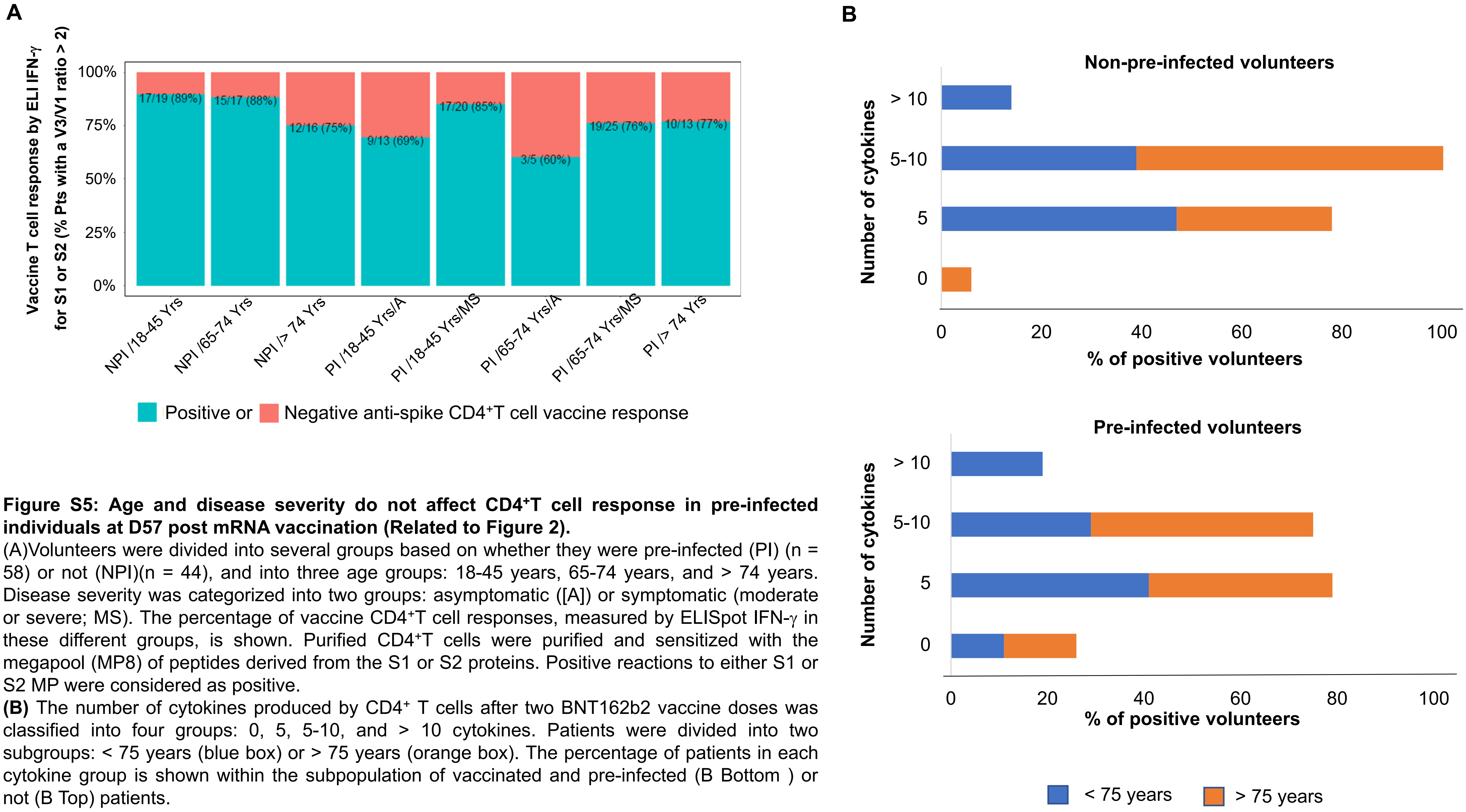

Figure S5: Age and disease severity do not affect CD4⁺T cell response in pre-infected individuals at D57 post mRNA vaccination (Related to Figure 2).

(A)Volunteers were divided into several groups based on whether they were pre-infected (PI) (n = 58) or not (NPI)(n = 44), and into three age groups: 18-45 years, 65-74 years, and > 74 years. Disease severity was categorized into two groups: asymptomatic ([A]) or symptomatic (moderate or severe; MS). The percentage of vaccine CD4⁺T cell responses, measured by ELISpot IFN-γ in these different groups, is shown. Purified CD4⁺T cells were purified and sensitized with the megapool (MP8) of peptides derived from the S1 or S2 proteins. Positive reactions to either S1 or S2 MP were considered as positive.

(B) The number of cytokines produced by CD4⁺ T cells after two BNT162b2 vaccine doses was classified into four groups: 0, 5, 5-10, and > 10 cytokines. Patients were divided into two subgroups: < 75 years (blue box) or > 75 years (orange box). The percentage of patients in each cytokine group is shown within the subpopulation of vaccinated and pre-infected (B Bottom) or not (B Top) patients.

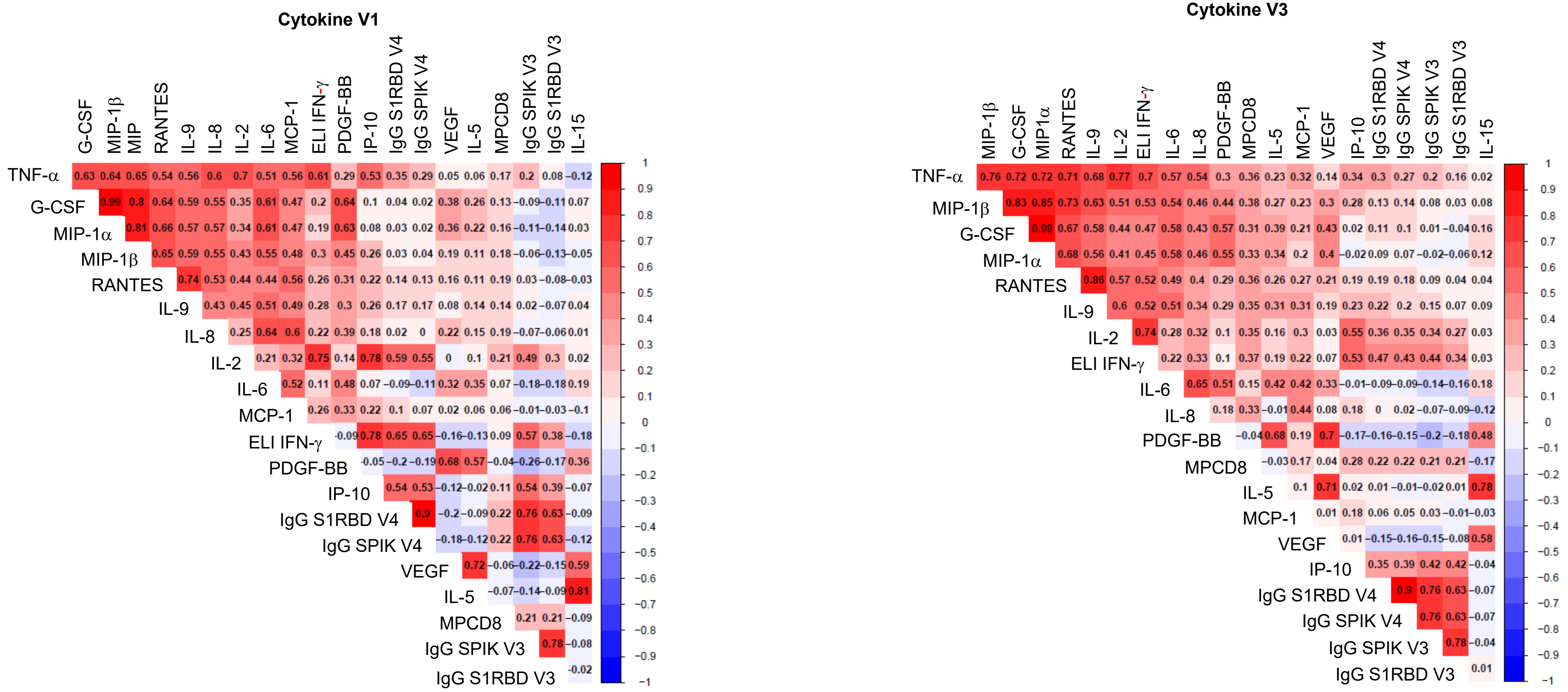

Figure S6: Correlation among various cytokines produced by CD4⁺T cells before (V1) and after (V3) vaccine administration and humoral responses (Related to Figure 4).

Non-parametric Spearman test for the analysis of correlation matrix illustrates the strength of the correlation between each cytokine produced by CD4⁺T cells before (V1) (Left) and following the vaccine administration at 3 months (V3) (Right) and the serological response in all patients (n = 128). The concentration of anti-spike or RBD IgG antibodies at 3 (V3) and 6 months (V4) is incorporated into this correlation matrix as a surrogate markers of serology. The scale for the correlation is shown at the right side of the matrix. Negative correlations are depicted in blue, while positive correlations are shown in red.

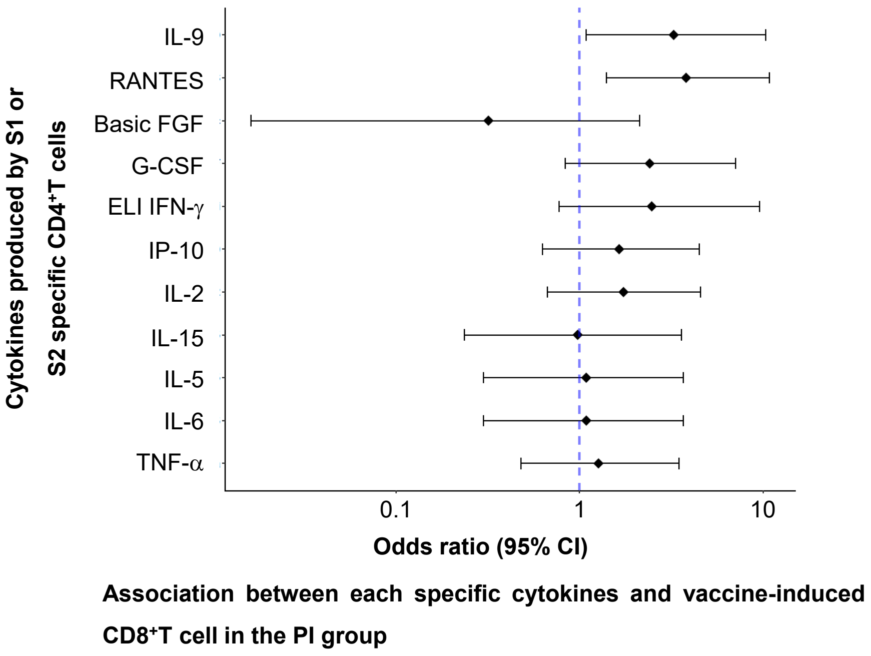

Figure S7: Cytokine profile detection derived from CD4$^+$T cells and vaccine-induced CD8$^+$T cells (Related to Figure 6).

A correlation was sought between the positivity (defined by the V3/VA ratio) of each cytokine produced by CD4$^+$T cells sensitized by S1 and S2 following vaccination (D57) in pre-infected volunteers (n = 58) and the concurrent induction of CD8$^+$T cell specific to S1 or S2. The Cox statistical test was used for the analysis.

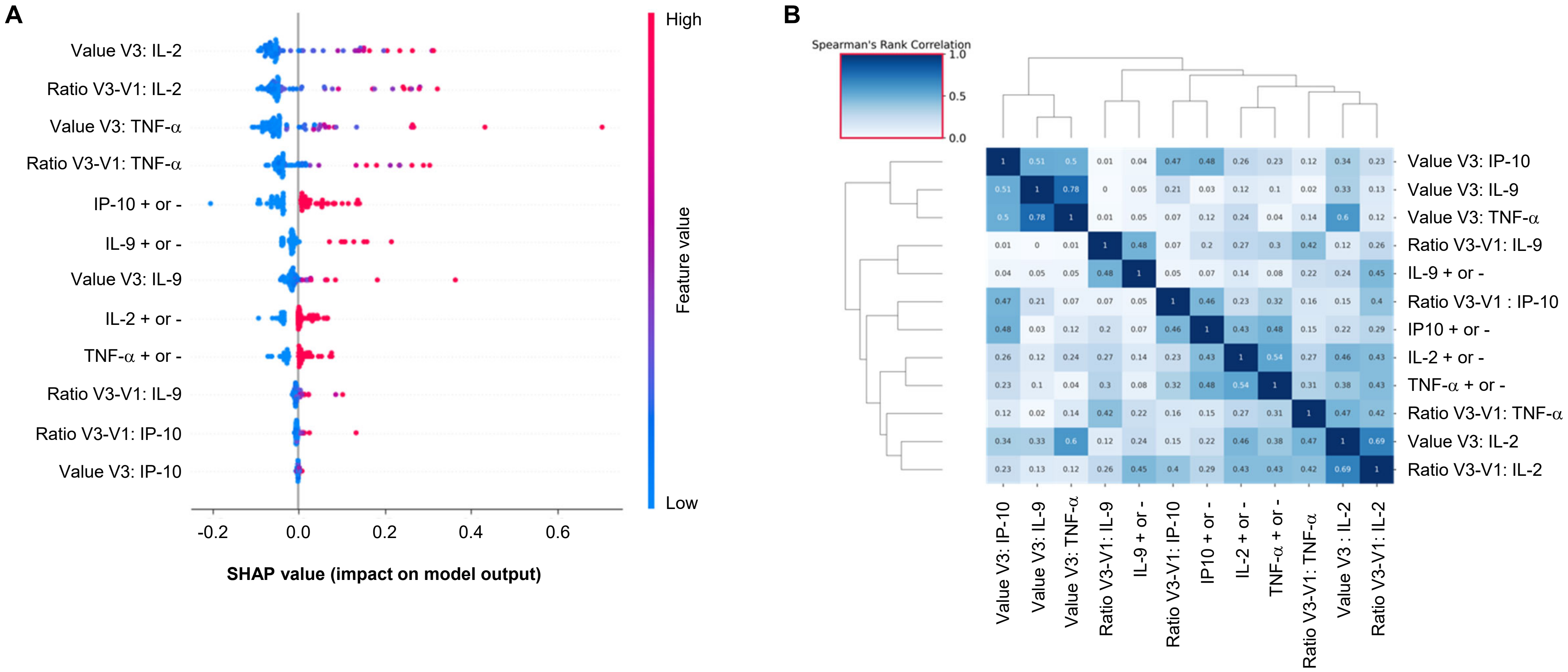

Figure S8: A Shapley additive (SHAP) analysis for the machine learning model setup in this study (related to Figure 6).

(A) The impact of each parameter (V3/V1 ratio, absolute value at V3, positivity or negativity of the test for each cytokine) on the classification is shown through a swarm plot of their SHAP values and distribution. The color of the dots represents the relative value of the feature in the Moderna dataset, with high values shown in red and low values in blue. The horizontal location of the dots indicates whether the effect of that feature value contributed positively or negatively to that prediction instance (x-axis).

(B) A heatmap displays the Spearman correlation coefficients obtained between the features, where dark blue corresponds to a Spearman correlation of 1 and white corresponds to a Spearman correlation of 0. Attached to the heatmap is a dendrogram that illustrates the hierarchies of feature clusters determined through agglomerative hierarchical clustering analysis.

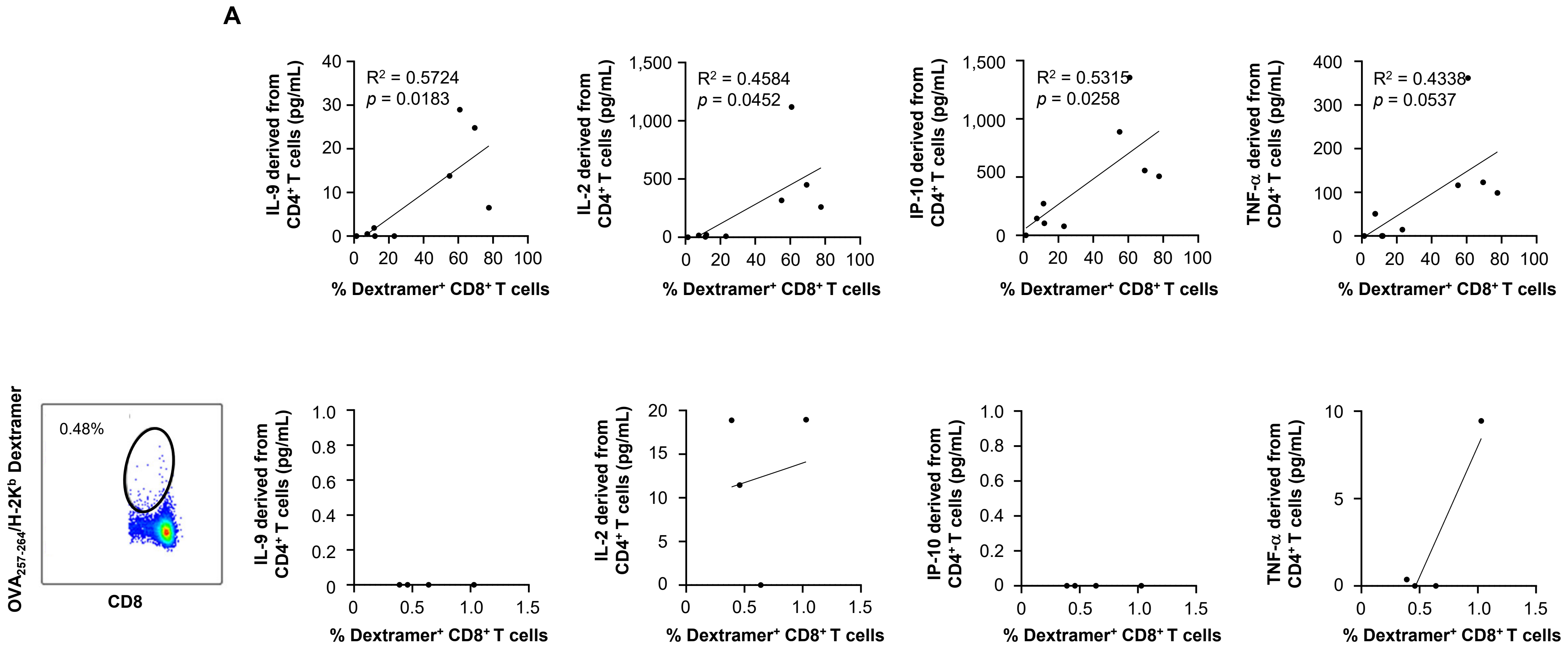

Figure S9: Correlation between the cytokine signature of CD4⁺T cells and the percentages of ovalbumin (OVA)-specific CD8⁺T cells in the context of high or low frequency of dextramer⁺ positive CD8⁺T cells (Related to Figure 6).

Mice (n = 4) were intranasally immunized as described in Figure 7. OVA (100 µg) was administered alone or combined with the adjuvant C-di-GMP (10 µg) **(A)** or with STxB-OVA without adjuvant (20 µg) **(B)**. After 21 days, concentrations of IL-9, IL-2, TNF-α, and IP-10 were measured in the supernatants of CD4⁺T cells sensitized for 36 hours with the $OVA_{265\text{-}280}$ peptide, which is restricted by CMHII. The Pearson statistical test was used for the analysis.

Age group, years	PI	NPI
18-45	33	19
65-74	30	17
> 74	13	16

Table S1: Distribution of pre-infected (PI) and non-pre-infected (NPI) patients in the Pfizer Cohort (Related to Figure 1 and 2).
Out of 145 participants, 17 were excluded for various reasons: i/ positive IgG-N at any visit until D57 (n = 3), ii/ not analyzed by ELISpot (n = 6), iii/ not analyzed by Luminex (n = 8). Thus, 128 patients were included in this study.
The average time between the date of the positive test (Infection) and the first vaccination for the PI patients was 9.3 months [7,2; 12,2].

The V3/V1 ratio for each cytokine

Cytokine	Median<br>N = 128	[Q1; Q3]
IL-15	1.00	[1.00; 1.34]
IL-5	1.00	[1.00; 1.62]
PDGF-BB	1.00	[0.77; 2.36]
IL-9	1.12	[0.88; 2.22]
IL-6	1.27	[0.75; 2.33]
VEGF	1.00	[0.85; 1.80]
G-CSF	1.39	[0.92; 1.98]
MIP-1b	1.37	[1.04; 1.86]
MCP-1	1.14	[0.93; 2.02]
MIP-1a	1.48	[0.99; 2.30]
RANTES	1.03	[1.00; 3.56]
IL-8	1.00	[0.83; 3.95]
IL-2	2.69	[1.42; 6.65]
TNFa	2.80	[1.50; 6.31]
IP-10	3.99	[1.42; 17.99]
ELI IFNγ	4.53	[2.28; 10.46]

Table S2: Type of cytokines inducing a vaccine CD4^{+}T cell response following BNT162b2 vaccination (Related to Figure 1 and 2).
A total of 128 volunteers were vaccinated, and T cell responses were measured as shown in Figure 1.
The median induction factor for vaccine response is depicted for each cytokine between V3 and V1. A response factor of at least 2 between V3 and V1 was required to define a T cell vaccine response. The red box highlights for which cytokines a vaccine T cell response (median fold change $\geq$ 2) was observed in over half of the volunteer population. All cytokines were quantified using Luminex, except for IFN-γ, which was assed by ELISpot.
